# Barriers to and facilitators of physical activity in adults living with and beyond cancer, with special emphasis on head and neck cancer: a systematic review of qualitative and mixed methods studies

**DOI:** 10.1007/s00520-023-07925-x

**Published:** 2023-07-17

**Authors:** Hannah C. Doughty, Ruaraidh A. Hill, Andrew Riley, Adrian W. Midgley, Joanne M. Patterson, Lynne M. Boddy, Simon N. Rogers, Michelle Maden, Nefyn H. Williams

**Affiliations:** 1 Department of Primary Care and Mental Health, University of Liverpool, Liverpool, L69 3GL UK; 2 Department of Health Data Science, University of Liverpool, L69 3GL Liverpool, UK; 3 Department of Sport and Physical Activity, Edge Hill University, Ormskirk, L39 4QP UK; 4 Liverpool Head and Neck Centre, University of Liverpool, L69 3GB Liverpool, UK; 5 The Physical Activity Exchange, Research Institute for Sport and Exercise Sciences, Liverpool John Moores University, Liverpool, L3 2EX UK; 6 Head and Neck Centre, Wirral University Teaching Hospital, Wirral, CH49 5PE UK

**Keywords:** Adherence, Behaviour change, Exercise, Oncology, Promotion

## Abstract

**Purpose:**

Physical activity can improve health outcomes for cancer patients; however, only 30% of patients are physically active. This review explored barriers to and facilitators of physical activity promotion and participation in patients living with and beyond cancer. Secondary aims were to (1) explore similarities and differences in barriers and facilitators experienced in head and neck cancer versus other cancers, and (2) identify how many studies considered the influence of socioeconomic characteristics on physical activity behaviour.

**Methods:**

CINAHL Plus, MEDLINE, PsycINFO, Scopus and Cochrane (CDSR) were searched for qualitative and mixed methods evidence. Quality assessment was conducted using the Mixed Methods Appraisal Tool and a Critical Appraisal Skills Programme Tool. Thematic synthesis and frequency of reporting were conducted, and results were structured using the Capability-Opportunity-Motivation-Behaviour model and Theoretical Domains Framework.

**Results:**

Thirty qualitative and six mixed methods studies were included. Socioeconomic characteristics were not frequently assessed across the included studies. Barriers included side effects and comorbidities (*physical capability*; *skills*) and lack of knowledge (*psychological capability*; *knowledge*). Having a dry mouth or throat and choking concerns were reported in head and neck cancer, but not across other cancers. Facilitators included improving education (*psychological capability*; *knowledge*) on the benefits and safety of physical activity.

**Conclusion:**

Educating patients and healthcare professionals on the benefits and safety of physical activity may facilitate promotion, uptakeand adherence. Head and neck cancer patients experienced barriers not cited across other cancers, and research exploring physical activity promotion in this patient group is required to improve physical activity engagement.

**Supplementary information:**

The online version contains supplementary material available at 10.1007/s00520-023-07925-x.

## Introduction

Cancer is a leading cause of death worldwide and approximately one-third of cancer-related deaths are caused by physical inactivity and unhealthy lifestyle behaviours [1]. Cancer survival is increasing [2], and there is an increase in the number of individuals undergoing invasive treatments and living longer with the consequences of cancer and its treatments. People living with and beyond cancer are at an increased risk of developing other chronic health conditions, and cancer survivors remain at a higher risk of their cancer recurring [3, 4]. Physical activity can reduce treatment-related symptoms, decrease the development of co-morbid conditions, reduce the chance of disease recurrence and improve overall quality of life [5, 6]. Although research supports the safety of physical activity during all treatment stages [7], physical activity levels often decrease during treatment and do not improve after treatment completion [8]. Patients living with and beyond cancer can suffer from a variety of physical and psychological side effects during and post cancer treatment [9] that can impede their ability to be physically active. In particular, head and neck cancer is one of the most debilitating cancers, as patients can experience difficulty breathing (dyspnoea), swallowing (dysphagia) and eating [10], and appearance-related concerns, which are either not apparent, or less severe in patients with other cancers. It is reported that only 30% of cancer patients meet recommended physical activity levels of at least 150 minutes of moderate-to-vigorous intensity aerobic activity per week [11], and 9% of head and neck cancer patients are reported to meet these recommendations [12]. However, research exploring barriers to and facilitators of physical activity in head and neck cancer is limited, and systematic reviews exploring this topic area have not included studies that have investigated head and neck cancer.

Socioeconomic factors are influential determinants of physical activity levels [13]; however, literature suggests that many studies do not consider the interactions between socioeconomic factors and physical activity in cancer patients [14]. Physical activity interventions are more likely to be successful if they are designed using theoretical underpinnings [15]. Using behaviour change theory to systematically identify barriers to and facilitators of physical activity in patients living with and beyond cancer may help to understand the determinants that influence physical activity promotion and behaviour. There have been previous systematic reviews that have explored barriers to and facilitators of physical activity in patients with cancer [16–19]. However, none of these reviews have used behaviour change theory to explore barriers and facilitators, or to understand and apply findings to physical activity promotion and practice. This systematic review aimed to explore barriers to and facilitators of physical activity in patients living with and beyond cancer, using the Capability-Opportunity-Motivation-Behaviour (COM-B) model [20, 21] and the Theoretical Domains Framework (TDF) [22, 23]. Secondary aims were to (1) explore similarities and differences in barriers and facilitators experienced in head and neck cancer versus other cancers and (2) identify how many studies considered the influence of socioeconomic characteristics on physical activity behaviour.

## Methods

This review was conducted using internationally recognised methods for systematic reviews, and is reported according to the Preferred Reporting Items for Systematic Reviews and Meta-Analyses (PRISMA) Guidelines [24] (see Online Resource 1 for PRISMA checklist). A protocol was registered with PROSPERO in September 2021 (record number: CRD42021277345).

### Search strategy

Searches were conducted across CINAHL Plus, MEDLINE, PsycINFO, Scopus and Cochrane (CDSR), for articles published from 1 January 2005 to 10 October 2022. The decision to search from 2005 onwards was to cover at least 15 years’ worth of literature in the context of current health service provision. Backward reference searching was conducted using reference lists of relevant systematic reviews identified, and by searching the reference lists of eligible studies. An information specialist assisted with the development of search terms and search implementation (MM). Searches were undertaken using a combination of Boolean operators, MeSH terms, and free-text terms including ‘cancer’, ‘neoplasm’, ‘physical activity’, ‘exercise’, ‘barrier’ and ‘facilitator’ (see Online Resource 2 for the full search strategy).

### Study selection

Peer-reviewed papers published between 1 January 2005 and 10 October 2022 were returned in the search. EndNote X9 reference manager for MacOS (Clarivate Analytics) was used to store references, and the online reviewing system Rayyan (Qatar Computing Research Institute) was used to aid the screening process. Duplicate records were identified using two electronic systems (EndNote and Rayyan) and by hand searching. Duplicate records were checked and removed. All titles and abstracts were screened by two independent reviewers (HD, AR/RH) and full text screening was conducted by authors HD and AR. A primary reason for exclusion was recorded at the full text stage. Any discrepancies on eligibility decisions were resolved through consensus and sometimes by consulting a third reviewer (NW/RH). Physical activity is defined as ‘any bodily movement produced by skeletal muscles, that results in energy expenditure’ [25]*.* Exercise is defined as a ‘subset of physical activity that is planned, structured, and repetitive’ [25]. Physical activity is used in the present review as a synonym of exercise. For the purpose of this review, a barrier was defined as a factor that impedes patients’ abilities to be physically active, or impedes healthcare professionals’ abilities to promote physical activity, and could include demographic, physical, social, or environmental factors [26]. A facilitator was defined as a factor that enables patients to be physically active, or enables healthcare professionals to promote physical activity, and could include demographic, physical, social, or environmental factors [26] (see Table 1 for eligibility criteria).

**Table 1:** Eligibility criteria

Inclusion criteria	Exclusion criteria
• Adults (≥ 18 years old) who had been diagnosed with any cancer type, and were at any stage of treatment • Family members/caregivers or health professionals involved in the care of cancer patients	• Patients with incurable disease (metastatic/palliative), or health professionals working predominantly in palliative care
• Qualitative, or mixed methods evidence available in English	• Quantitative studies, conference abstracts, case studies, or editorial articles
• Barriers to, or facilitators of, physical activity promotion or participation (one of the main outcomes of the study)	• Barriers to, or facilitators of, a physical activity intervention, or specific to those with long-term conditions other than cancer • Patients who had participated in a previous physical activity programme or intervention • Additional interventions or health promotion advice (such as nutritional or smoking cessation advice)

### Quality assessment of included studies

The quality of included studies were independently assessed by authors HD and AR using the *Mixed Methods Appraisal Tool* (MMAT) [27]. Question 4 from the *Critical Appraisal Skills Programme* qualitative study assessment checklist [28] was appended as an additional question to assess recruitment strategies across the included studies. Question 4 was used as the MMAT includes a similar sampling strategy question for quantitative (descriptive) studies, but not for qualitative or mixed methods studies. The MMAT consists of two screening questions and five methodological questions with response options of ‘yes’, ‘no’ or ‘can’t tell’. MMAT (Version 18) does not provide an overall score from the ratings of each criterion but enables specific strengths and limitations to be critically evaluated.

### Data extraction and data synthesis

A data extraction tool was created in Microsoft Excel and Microsoft Word (Microsoft Corporation, USA) and extracted study characteristics included study author, title, aim, sample size, population, outcome measure, type of activity and country. Clinical characteristics included cancer type and length of time from diagnosis (see Online Resource 3 for the study characteristics table). Socioeconomic characteristic reporting were assessed using the PROGRESS-plus equalities domains [29]. This tool is comprised of equality domains understood to influence health opportunities, including the opportunity to participate in and benefit from physical activity. Data were categorised as either collected, analysed or discussed. Data were characterised as ‘collected’ if relevant data were tabulated or summarised; data were characterised as ‘analysed’ if data were either included in the results or analysis section of the study, or included in any form of statistical analysis, or within the qualitative themes. Lastly, data were characterised as ‘discussed’ if data were described within the discussion section of the study, or if there was mention of any implications related to a particular characteristic.

**Fig. 1:**
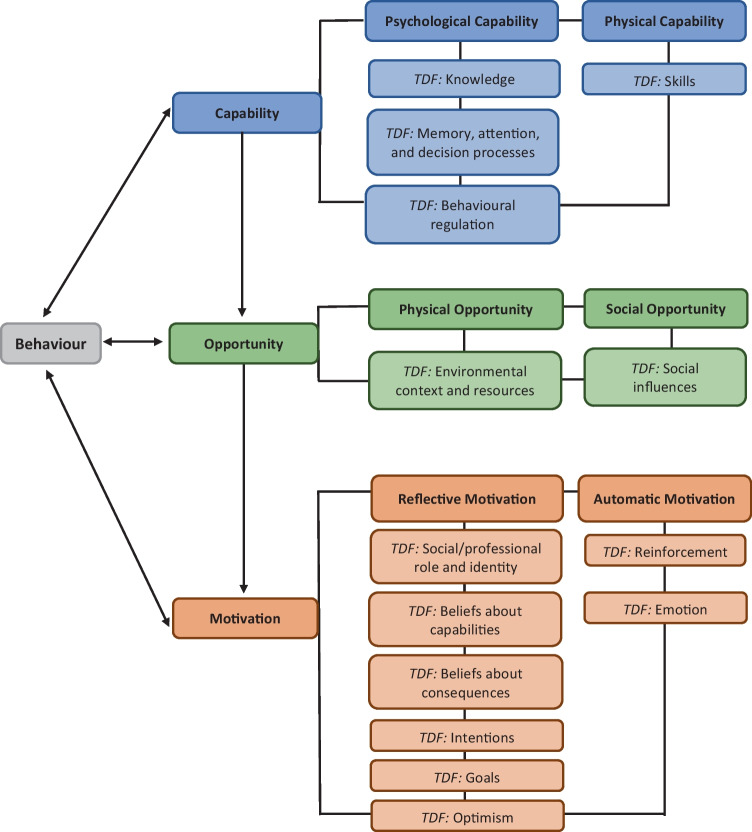
Capability-Opportunity-Motivation-Behaviour (COM-B) [20, 21] and Theoretical Domains Framework (TDF) [22, 23] Behaviour Change Domains (adapted from [80])

Data were analysed and synthesised using a data-driven bottom-up approach, and Thomas and Harden’s method of thematic synthesis [30] was used to inductively code, develop and generate themes from the barriers and facilitators identified across the included studies. Inductive data-driven themes were deductively categorised using the relevant domains of the COM-B model [20, 21] and the TDF [22, 23]. The COM-B model posits that behaviour change is dependent upon an individual possessing the capability, opportunity and motivation in order to change their behaviour [20, 21]. The TDF builds on the COM-B model and consists of 14 domains that aim to further understand the underlying barriers to and facilitators of evidence-based behaviour change [22, 23] (see Fig. 1). Frequency of reporting was used to establish how frequently the barriers and facilitators were discussed across the included studies (see Tables 2, 3 and 4), and has been used to synthesise findings in a previous scoping review [31]. Comparisons were made to resolve discrepancies until the final themes were confirmed (HD, AR, NW, RH).

**Table 2:** Barriers to physical activity identified across the included studies (*n* = 36)

Themes	Frequency	Citation(s)
Capability-related barriers		
C1: Physical capability		
*C1.1 Physical ability (side effects/symptoms) (TDF domain skills)*		
C1.1.1: Fatigue	22	[32–36, 38, 40, 42–44, 46–52, 54, 60, 61, 65, 66]
C1.1.2: Comorbidities (*such as joint pain or injuries*; *back pain*; *arthritis*; *lung problems*; *heart disease*, *prior injury and neuropathy*)	18	[32, 34, 39, 40, 43–48, 50–53, 57, 60, 64, 65]
C1.1.3: Too unwell/general side effects	15	[32, 35, 37, 44–46, 50, 52, 57, 59, 62–65, 67]
C1.1.4: Pain	13	[32–34, 37, 39, 41, 42, 45–48, 60, 65]
C1.1.5: Age	9	[34, 39, 40, 42, 43, 45, 46, 48, 56]
C1.1.6: Incontinence	7	[34, 35, 40, 44, 45, 53, 65]
C1.1.7: Limited fitness capacity	7	[38, 39, 49, 51, 55, 60, 66]
C1.1.8: Muscle wastage/lack of strength	7	[33, 36–38, 42, 44, 52]
C1.1.9: Gastric symptoms (nausea/vomiting/diarrhoea/constipation)	5	[33, 37, 45, 54, 65]
C1.1.10: Complex patient group	2	[55, 64]
C1.1.11: Insomnia	2	[33, 45]
C1.1.12: Anaemia	1	[45]
C1.1.13: Catheter	1	[39]
C1.1.14: Difficulty breathing (dyspnoea)	1	[33]
C1.1.15: Drooling	1	[33]
C1.1.16: Intravenous chemotherapy device	1	[66]
C1.1.17: Loss of appetite	1	[33]
C1.1.18: Ostomy	1	[66]
C1.1.19: Physical ability and reception to physical activity advice	1	[55]
C1.1.20: Sexual issues	1	[38]
C1.1.21: Vertigo	1	[45]
C1.1.22: Weight gain	1	[60]
C1.1.23: Tingling in fingers and feet	1	[54]
*C1.2 Lifestyle (TDF domain skills)*		
C1.2.1: Prior activity or experience (inactivity)	1	[57]
C2: Psychological capability		
*C2.2 Lack of understanding regarding the importance of physical activity and how to promote it (TDF domain knowledge)*		
C2.2.1: Lack of knowledge regarding physical activity or how much activity to do	12	[34, 35, 39, 49, 52–54, 60, 61, 64, 66, 67]
C2.2.2: Lack of understanding regarding the potential benefits or importance	6	[42, 57, 60, 61, 63, 65]
C2.2.3: Lack of knowledge regarding how to promote physical activity	4	[56–58, 63]
C2.2.4: Lack of consensus between professionals	4	[47, 55, 56, 67]
C2.2.5: Lack of education, training, or evidence	3	[55–57]
C2.2.6: Awareness of need for physical activity programmes	2	[60, 63]
C2.2.7: Healthcare professionals own perception of physical activity	2	[55, 64]
C2.2.8: Misunderstanding from professionals regarding physical health	1	[65]
C2.2.9: Lack of knowledge about cancer	1	[67]
C2.2.10: Psychological ability and reception to physical activity advice	1	[55]
Opportunity-related barriers		
O1: Physical opportunity		
*O1.1 Lack of time to participate in or to promote physical activity (TDF domain environmental context and resources)*		
O1.1.1: Lack of time	24	[32–35, 40–42, 44–47, 51–53, 55–62, 64, 65]
O1.1.2: Lack of information provision or resources	16	[32, 34, 35, 39, 40, 47–49, 51, 53, 56, 58, 60, 63, 65, 66]
O1.1.3: Lack of service support or specialists	6	[55–57, 61–63]
O1.1.4: Healthcare professionals forgot to discuss physical activity	2	[61, 64]
*O1.2 Unmet needs (TDF domain environmental context and resources)*		
O1.2.1: Personalised programmes	3	[54, 60, 63]
O1.2.2: Group activities	1	[65]
*O1.3 Environmental factors (TDF domain environmental context and resources)*		
O1.3.1: Availability of, or access to, facilities or programmes	15	[32, 36, 39, 42, 44, 45, 50, 51, 55, 58, 60, 62–64, 67]
O1.3.2: Weather	7	[32, 42, 43, 48–50, 64]
O1.3.3: Wildlife concerns	1	[36]
O2: Social opportunity	Frequency	Citation(s)
*O2.1: Socioeconomic factors (TDF domain social influences)*		
O2.1.1: Financial constraints, travel requirements, or work obligations	17	[32–35, 38, 40, 48, 50, 51, 53, 56, 60–64, 67]
O2.1.2: Family responsibilities/gatekeeping, or social obligations	8	[32, 39, 42, 48, 51–53, 66]
O2.1.3: Lack of company or social support	8	[32, 42, 43, 48, 50, 51, 54, 57]
O2.1.4: Cultural responsibilities/appropriateness or community implications	3	[42, 43, 50]
O2.1.5: Crime (being active outdoors)	3	[36, 42, 51]
*O2.2 Healthcare professional influences (TDF domain social influences)*		
O2.2.1: Healthcare professionals don’t want to jeopardise relationship with patient	1	[58]
Motivation-related barriers		
M1: Automatic motivation		
*M1.1: Psychological barriers (TDF domain emotion)*		
M1.1.1: Self-conscious	11	[34, 35, 38, 47, 50, 52, 54, 60, 62, 64, 66]
M1.1.2: Lack of motivation	9	[32, 33, 40, 45, 46, 51, 52, 57, 60]
M1.1.3: Psychological distress/managing expectations	8	[38, 41, 42, 45, 52, 57, 58, 64]
M1.1.4: Lack of confidence/self-efficacy	7	[32, 34–38, 60]
M1.1.5: Anxiety or depression	6	[42, 46, 52, 57, 61, 64]
M1.1.6: Lack of enjoyment or interest	4	[37, 57, 60, 65]
M1.1.7: Previous negative experience or negative attitude toward physical activity	3	[37, 39, 64]
M1.1.8: Impact of cancer diagnosis/patient mindset	3	[35, 45, 61]
M1.1.9: Difficulty keeping spirits up	1	[39]
M2: Reflective motivation		
*M2.1: Fear of harm (TDF domain beliefs about consequences)*		
M2.1.1: Concerns over the general safety of being physically active; concerns over symptoms/encouraging rest or conflicting messages	19	[32, 34, 35, 39, 47, 48, 50–52, 54–57, 60–62, 64, 66, 67]
M2.1.2: Fear of overexertion or feeling the need to conserve energy for treatment	5	[42, 52, 58, 64, 67]
M2.1.3: Fear of injury/falling	4	[43, 45, 47, 55]
M2.1.4: Fear of judgement	3	[35, 38, 54]
M2.1.5: Fear of germs or infection	1	[42]
M2.1.6: Fear of feeling nauseous	1	[39]
M2.1.7: Fear of fainting	1	[39]
M2.1.8: Fear of the unknown	1	[39]
M2.1.9: Fear of disproval from family or friends	1	[37]
M2.1.10: Fear of being unable to be active	1	[45]
*M2.2: Perceptions of a patient’s ability (TDF domain beliefs about capabilities)*		
M2.2.1: Perceptions of interest or ability	2	[57, 58]
*M2.3: Confidence (TDF domain beliefs about capabilities)*		
M2.3.1: Lack of confidence in own ability	4	[34, 39, 60, 64]
*M2.4: Lifestyle factors (TDF domain goals and intentions)*		
M2.4.1: Not a priority or lack of discipline	10	[48, 50, 55, 57–59, 63, 64, 66, 67]
M2.4.2: Self-perceived as active enough	4	[42, 48, 50, 65]
*M2.5: Role to discuss physical activity (TDF domain social/professional role and identity)*		
M2.5.1: Professional role to promote physical activity	2	[55, 64]

**Table 3:** Barriers to physical activity identified as specific to head and neck cancer (*n* = 1)

Themes	Frequency	Citation(s)
Capability-related barriers		
C1: Physical capability		
*C1.1 Treatment-related side effects (TDF domain skills)*		
C1.1.1: Dry mouth or throat	1	[46]
Opportunity-related barriers		
O1: Physical opportunity	Frequency	Citation(s)
*O1.1 Environmental factors (TDF domain environmental context and resources)*		
O1.1.1: Weather exacerbating symptoms	1	[46]
Motivation-related barriers		
M2: Reflective motivation	Frequency	Citation(s)
*M1.1: Fear of harm (TDF domain beliefs about consequences)*		
M1.1.1: Choking concerns	1	[46]

**Table 4:** Facilitators of physical activity identified across included studies (*n* = 34)

Themes	Frequency	Citation(s)
Capability-related facilitators		
C1: Physical capability		
*C1.1 Ability to be active (TDF domain skills)*		
C1.1.1: No side effects/ability to be active	3	[45, 46, 65]
C1.1.2: Physical ability and reception to physical activity advice	2	[33, 55]
*C1.2 Lifestyle (TDF domain skills)*		
C1.2.1: Prior activity levels	6	[35, 42, 45, 47, 64, 65]
C2: Psychological capability		
*C2.1 Knowledge of importance (TDF domain knowledge)*		
C2.1.1: Awareness of benefits or know how to promote physical activity	9	[42, 47, 49, 50, 53, 59–61, 65]
C2.1.2: Psychological ability and reception to physical activity advice	1	[55]
C2.1.3: Healthcare professionals own perception of physical activity	1	[55]
*C2.2 Education (TDF Domain knowledge)*		
C2.2.1: Increased education, information provision and resources	17	[36, 39, 40, 45, 47, 50, 51, 55–61, 63–65]
Opportunity-related facilitators		
O1: Physical opportunity		
*O1.1 Importance of physical activity promotion and support (TDF domain environmental context and resources)*		
O1.1.1: Healthcare professional support or promotion	12	[33–36, 45, 52, 53, 59–62, 65]
O1.1.2: Tailored support or modified activities	10	[35, 40, 41, 51, 52, 54, 61, 62, 65, 67]
*O1.2 Integration of physical activity promotion (TDF domain environmental context and resources)*		
O1.2.1: Integration of physical activity promotion into routine service delivery/additional healthcare professionals or policies to support promotion	9	[33, 50, 55, 57, 58, 60, 61, 63, 64]
*O1.3 Environmental support (TDF domain environmental context and resources)*		
O1.3.1: Environmental motivators (access to green space/scenic surroundings)	5	[34, 36, 42, 45, 67]
O1.3.2: Ability to attend/access to facilities or information	4	[49, 51, 55, 60]
O1.3.3: Close to home or ability to do at home	3	[33, 42, 65]
O2: Social opportunity	Frequency	Citation(s)
*O2.1: Socioeconomic support (TDF domain social influences)*		
O2.1.1: Social support	20	[32, 34, 35, 37, 40, 42, 43, 47–50, 52, 54, 57, 60–63, 65, 67]
O2.1.2: Financial support	3	[50, 65, 67]
O2.1.3: Cultural traditions/religious faith	3	[43, 50, 67]
O2.1.4: Community involvement	1	[51]
Motivation-related facilitators		
M1: Automatic motivation		
*M1.1: Physical benefits (TDF domain reinforcement)*		
M1.1.1: Feeling or perceiving there are benefits (including: improvements to quality of life, pain, fatigue, functional capability, weight management, survivorship and being fitter for treatment)	19	[32–38, 40, 41, 43, 45, 46, 48, 52, 55, 61–63, 67]
*M1.2: Psychological (TDF domain emotion)*		
M1.2.1: Coming to terms with symptoms	1	[38]
*M1.3: Physical reinforcement (TDF domain reinforcement)*		
M1.3.1: Electronic monitoring and reinforcement	2	[43, 50]
*M1.4: Enjoyment (TDF domain emotion)*		
M1.4.1: Interest or enjoyment in participating	5	[32, 41, 45, 52, 67]
M1.4.2: Previous positive experience	2	[32, 39]
M1.4.3: Desire to seek out information	1	[49]
M2: Reflective motivation	Frequency	Citation(s)
*M2.1: Perceptions of own ability (TDF domain beliefs about capabilities)*		
M2.1.1: Self-efficacy	2	[48, 50]
*M2.2: Perceptions of patient interest or ability (TDF domain beliefs about capabilities)*		
M2.2.1: Perceptions of those able or interested in participating	1	[58]
*M2.3: Goal setting (TDF domain goals and intentions)*		
M2.3.1: Integrated into lifestyle	9	[32, 41, 43, 45, 47, 48, 51, 52, 65]
M2.3.2: Diagnosis as a teachable moment/taking control	4	[33, 35, 39, 62]
M2.3.3: Improve psychological/physical health	3	[34, 46, 62]
M2.3.4: Goal setting	3	[36, 39, 61]
M2.3.5: Return to normality	2	[52, 53]
M2.3.6: Creating habits	1	[42]
M2.3.7: Desire to be more active post diagnosis	1	[49]

## Results

**Fig. 2:**
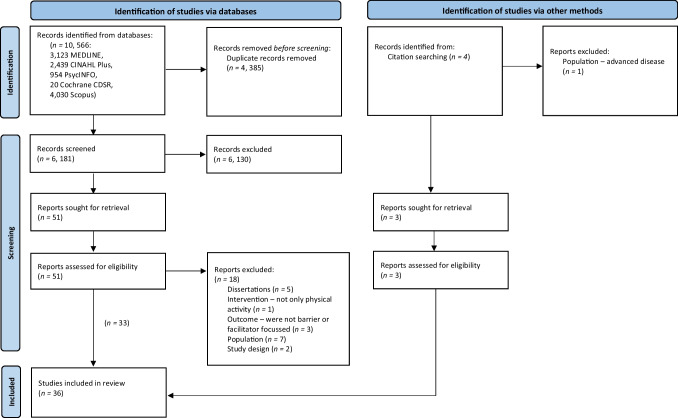
PRISMA 2020 flow diagram of databases and citation searching for the identification, screening, eligibility and inclusion of studies [24]

After the removal of duplicate references, the titles and abstracts of 6181 studies were screened, of which 51 met the criteria for further review. The full texts of 51 records were screened, resulting in 33 studies meeting the inclusion criteria. An additional four studies were identified through backward searching, resulting in the identification of an additional three eligible studies and a total of 36 included studies (see Fig. 2).

### Study characteristics

Thirty qualitative and six mixed method studies published between September 2008 and September 2022 were included. Following critical appraisal, all studies were of sufficient methodological quality to remain included (see Online Resource 4). Twenty-three studies included patients only [32–54]; 10 studies included healthcare professionals [55–64]; two studies included patients, caregivers and healthcare professionals [65, 66]; and one study included patients and family members/friends [67]. Qualitative studies involved interviews (*n* = 18) [32, 35–38, 41, 44, 45, 49, 58–66], focus groups (*n* = 7) [40, 42, 50, 51, 53, 55, 56] or a mixture of the two methods (*n* = 5) [39, 43, 52, 57, 67]. Five mixed methods studies included questionnaires and interviews/focus groups [33, 34, 46, 47, 54], and one included questionnaires, interviews and accelerometer data [48]. Ten studies incorporated multiple cancer types [33, 35, 37, 49, 55, 60–63, 67], and other studies were specific to breast cancer (*n* = 11) [32, 41–43, 47, 50–52, 54, 56, 64]; prostate cancer (*n* = 6) [34, 38, 40, 44, 53, 59]; colorectal cancer (*n* = 3) [45, 65, 66]; breast, prostate and colorectal cancer (*n* = 2) [39, 58]; head and neck cancer (*n* = 2) [46, 48]; lung cancer (*n* = 1) [57]; and breast and kidney cancer (*n* = 1) [36]. Of the studies involving patients (*n* = 25), patients were either post-treatment (*n* = 14) [32–35, 41, 43, 44, 46, 48–52, 67], during and post-treatment (*n* = 5) [36, 38, 40, 42, 47], during treatment (*n* = 3) [37, 53, 66], pre-treatment (*n* = 1) [45], pre- and post-treatment (*n* = 1) [65] or pre- and during-treatment (*n* = 1) [39]. Only a small amount of data pertaining to the PROGRESS-plus equality domains were collected, analysed and discussed (see Online Resource 5). Studies were conducted in the USA (*n* = 7) [36, 43, 47, 50, 51, 53, 67], Canada (*n* = 5) [32, 56, 62–64], New Zealand (*n* = 4) [35, 44, 59, 60], the UK (*n* = 4) [38, 46, 49, 52], Australia (*n* = 3) [34, 40, 57], Sweden (*n* = 3) [39, 41, 45], the Netherlands (*n* = 2) [48, 65], France (*n* = 1) [37], Germany (*n* = 1) [58], Hong Kong (*n* = 1) [33], Italy (*n* = 1) [55], Malaysia (*n* = 1) [42], South Korea (*n* = 1) [54] and Spain (*n* = 1) [66], and one study was conducted with healthcare professionals working in Australia, Canada and the UK (*n* = 1) [61].

### Barriers

All of the barriers and facilitators identified were relevant to all of the COM-B constructs and to 11 of the 14 TDF domains (*skills*; *knowledge*; *environmental context and resources*; *social influences*; *beliefs about capabilities*; *beliefs about consequences*; *social/professionals role and identity*; *reinforcement*; *intentions*; *goals and emotion*).

### Capability-related barriers

Fatigue [32–36, 38, 40, 42–44, 46–52, 54, 60, 61, 65, 66] and pain [32–34, 37, 39, 41, 42, 45–48, 60, 65] were the most frequently cited treatment-related barriers to physical activity participation. Comorbidities (such as arthritis, lung and heart problems and neuropathy) were also frequently cited barriers [32, 34, 39, 40, 43–48, 50–53, 57, 60, 64, 65] (*physical capability*; TDF *skills*). Treatment-related side effects that were specific to head and neck cancer and impacted their ability to be physically active included having a dry mouth or throat [46]. Lack of understanding regarding the potential benefits of physical activity [42, 57, 60, 61, 63, 65] and patients’ lack of knowledge regarding what to do were identified as barriers by patients and healthcare professionals [34, 35, 39, 49, 52–54, 60, 61, 64, 66, 67] (*psychological capability*; TDF *knowledge*). Healthcare professionals identified a lack of knowledge with regard to how physical activity could be promoted to patients in-practice [56–58, 63].

### Opportunity-related barriers

Lack of time to participate in or to promote physical activity were identified as frequently reported barriers [32–35, 40–42, 44–47, 51–53, 55–62, 64, 65], and lack of information provision was identified as a barrier by patients and healthcare professionals [32, 34, 35, 39, 40, 47–49, 51, 53, 56, 58, 60, 63, 65, 66] (*physical opportunity*; TDF *environmental context and resources*). Access to and availability of facilities were identified as barriers by both patients and healthcare professionals [32, 36, 39, 42, 44, 45, 50, 51, 55, 58, 60, 62–64, 67]. Financial constraints, travel requirements, or work obligations [32–35, 38, 40, 48, 50, 51, 53, 56, 60–64, 67] were cited as barriers to physical activity by both patients and healthcare professionals (*social opportunity*; TDF *social influences*).

### Motivation-related barriers

Lack of motivation [32, 33, 40, 45, 46, 51, 52, 57, 60], lack of enjoyment or interest [37, 57, 60, 65], feeling self-conscious [34, 35, 38, 47, 50, 52, 60, 62, 64, 66] and lacking in confidence [32, 34–38, 60] were identified as barriers to physical activity participation (*automatic motivation*; TDF *emotion*). Concerns regarding the general safety of being physically active were frequently cited by healthcare professionals and patients [32, 34, 35, 39, 47, 48, 50–52, 54–57, 60–62, 64, 66, 67] (*reflective motivation*; TDF *beliefs about consequences*). Concerns included fear overexertion [42, 52, 58, 64, 67], fear of injury [43, 45, 47, 55] and fear of judgement [35, 38, 54]. Choking concerns [46] were identified in head and neck cancer, with one patient describing the need to always carry water.

### Facilitators

There were no facilitators identified across any included studies that were specific to head and neck cancer.

#### Capability-related facilitators

Prior activity levels were identified as a facilitator by patients and healthcare professionals [35, 42, 45, 47, 64, 65], and being physically active pre-diagnosis, influenced a patient’s ability to continue being physically active post-treatment (*physical capability*; TDF *skills*). Some patients reported not experiencing any treatment-related side effects [45, 46, 65], and feeling able to be physically active. Being aware of the benefits was cited as a facilitator to physical activity promotion and participation by patients, family members and healthcare professionals [42, 47, 49, 50, 53, 59–61, 65]. Knowledge of how to promote physical activity was identified as a facilitator by healthcare professionals [60]. Increasing education and providing clear information and resources [36, 39, 40, 45, 47, 50, 51, 55–61, 63–65] were identified as key facilitators by patients and healthcare professionals (*psychological capability*; TDF *knowledge*).

#### Opportunity-related facilitators

Patients, family members and healthcare professionals reported the importance of physical activity being approved and encouraged by healthcare professionals across services [33–36, 45, 52, 53, 59–62, 65] (*physical opportunity*; TDF *environmental context and resources*). Tailoring advice or resources to the individualised needs of cancer patients [35, 40, 41, 51, 52, 61, 62, 65, 67] and social support [32, 34, 35, 37, 40, 42, 43, 47–50, 52, 54, 57, 60–63, 65, 67] were identified as important facilitators by patients, family members and healthcare professionals. Some healthcare professionals perceived the support of fellow patients as important, although not all patients or family members shared this viewpoint [65]. Some professionals reported that many patients wanted to regain a sense of normality and preferred to access mainstream programmes, rather than cancer-specific ones [60] (*social opportunity*; TDF *social influences*).

#### Motivation-related facilitators

Feeling the benefits or perceiving that there are benefits to being physically active was identified as a motivator for patients [32–38, 40, 41, 43, 45, 46, 48, 52, 54, 55, 61–63, 67] (*automatic motivation*; TDF *reinforcement*). Such benefits included feeling as though being active was helping them to fight their cancer [35], and experiencing psychological improvements such as increased self-esteem, and feeling positive and more relaxed [34]. Some patients expressed interest or enjoyment in being physically active [32, 41, 45, 52, 67] (*automatic motivation*; TDF *emotion*), and some healthcare professionals reported that physical activity should be integrated into activities of daily living [32, 41, 43, 45, 47, 48, 51, 65] (*reflective motivation*; TDF *goals and intentions*).

## Discussion

### Summary of main findings

This review identified that treatment-related side effects can affect patients’ abilities to be physically active (*physical capability*; TDF *skills*). Lack of knowledge regarding the benefits of physical activity and lack of knowledge regarding what to do or what to advise were frequently reported barriers by both patients and healthcare professionals (*psychological capability*; TDF *knowledge*). Lack of time to promote or engage in physical activity (*physical opportunity*; *environmental context and resources*) and safety concerns (*reflective motivation*; TDF *beliefs about consequences*) were also frequently cited barriers by patients and healthcare professionals. Facilitators included improving education for patients, family members and healthcare professionals (*psychological capability*; TDF *knowledge*) on the benefits and safety of physical activity across the treatment trajectory (*reflective motivation*; TDF *beliefs about consequences*).

#### Comparison with previous literature

Consistent with previous literature [16], treatment-related side effects were identified as barriers to physical activity participation in the current review, and fatigue and pain were most frequently cited. Fatigue is highly prevalent in patients both during and post cancer treatment and persists at a higher-than-baseline level after treatment is completed, often for years [68]. Physical activity interventions have been shown to have the greatest improvement in reducing cancer-related fatigue, when compared with pharmacological treatments [69]. Consistent with previous literature, more than 50% of cancer patients receiving treatment reported pain [70], and pain continues to be a prevalent symptom in patients with cancer. Physical activity can be beneficial in reducing pain [71]; however, previous research has identified that 79% of patients reported a decrease in physical activity levels post-diagnosis [72]. Despite lack of research exploring barriers to and facilitators of physical activity in head and neck cancer, the current review identified barriers specific to head and neck cancer that were not cited across other cancer types. Treatment-related side effects that were specific to head and neck cancer included having a dry mouth or throat [46]. Previous research has reported that head and neck cancer patients ranked dry mouth or throat as the most challenging barrier, out of 36 different barriers to physical activity [10]. These symptoms significantly reduce head and neck cancer patients’ abilities to be physically active and support the need for head and neck cancer to be considered a distinct population within the context of physical activity [73]. Although previous reviews exploring barriers to and facilitators of physical activity did not cite comorbidities as a barrier, the current review identified that comorbidities were a frequently cited barrier to physical activity participation. Cross-sectional research has found that comorbidities were a significant negative predictor of physical activity levels across a variety of cancer types [74]. Physical activity has been shown to reduce the risk of developing comorbidities, such as reducing the risk of developing heart disease [75]. However, as patients with comorbidities struggle to be physically active, tailoring advice and activities to the needs of each patient is required.

The present review identified that lack of knowledge regarding the benefits of physical activity and lack of knowledge regarding what to do or what to advise were frequently reported barriers. These findings may help to explain why previous research has identified that some healthcare professionals did not feel confident promoting physical activity, and did not discuss the benefits with patients [76]. Conversely, a recent survey identified that 68% of healthcare practitioners reported that physical activity counselling was part of their routine practice [77]. Despite this, the current review identified that lack of time resulted in the inability for healthcare professionals to promote physical activity. Current evidence supports the safety of physical activity for cancer patients across all treatment stages [7]; however, consistent with previous literature exploring barriers to and facilitators of physical activity participation [17–19], fear of causing harm by being physically active was identified as a barrier in the current review. Additional fears not cited across previous reviews included fear of judgment [35, 38, 54], fear of germs or infection [42], fear of nausea [39], fear of fainting [39] and fear of the unknown [39]. Choking whilst being physically active was identified in head and neck cancer [46], and previous research has reported that 41% of head and neck cancer patients experienced excess mucus that caused choking [78], which may explain why this was a specific concern experienced within this patient group.

### Strengths and limitations

This is the first systematic review to use the COM-B [20, 21] and TDF [22, 23] to identify barriers to and facilitators of physical activity in patients living with and beyond cancer, with a special emphasis on head and neck cancer. Its strengths include the application of a rigorous dual screening process for the inclusion and quality assessment of all studies, reducing selection bias and potential error. Incorporating the views of patients, family members and healthcare professionals permitted a variety of perspectives to be collected within care pathways relating to cancer and physical activity. The fact that this review only included studies conducted in middle- to high-income countries and available in English is a limitation. Evidence published in other languages may have been missed and findings may not be applicable to low-income countries. Only the following databases (CINAHL Plus, MEDLINE, PsycINFO, Scopus and Cochrane [CDSR]) were searched using the selected search terms, and quantitative evidence and grey literature were not included; therefore, some potentially relevant studies may have been missed.

### Implications for practice and future research

These findings have important implications for practice. Although healthcare professionals promoting physical activity has been shown to have a direct impact on cancer patients physical activity levels [79], lack of time and lack of information or resources may prevent healthcare professionals promoting physical activity in practice. Improving education on the benefits and safety of physical activity across the treatment trajectory may improve physical activity promotion and participation. Secondly, despite this review identifying socioeconomic factors such as financial implications and social support as both a barrier to and facilitator of physical activity engagement, only a small amount of PROGRESS-plus equality domains [29] were collected, analysed and discussed. This supports the findings from previous literature [14], and indicates that the influence of socioeconomic factors on physical activity participation was not frequently assessed across the included studies. As socioeconomic factors are influential determinants of physical activity levels, both researchers and healthcare professionals need to consider their influence when designing physical activity programmes, referring patients to physical activity programmes, and providing physical activity advice.

## Conclusion

Despite the reported benefits of physical activity for patients living with and beyond cancer, this review identified a number of barriers to promotion and participation including side effects, comorbidities and lack of knowledge. Education and training on the benefits and safety of physical activity across the treatment trajectory should be provided to healthcare professionals, to enable them to promote physical activity to their patients. As head and neck cancer patients experienced specific barriers that impacted their ability to be physically active, exploring whether physical activity is promoted across health services for this patient group is required to understand how best to support practice. Our findings help to understand barriers to and facilitators of physical activity within a theoretical framework, which can be used to identify behaviour change techniques required to improve promotion, uptake and adherence, to reduce the detrimental effect of inactivity.

## Supplementary information

Below is the link to the electronic supplementary material.

Supplementary file1 (DOCX 102 KB) Supplementary information can be found in the attached PDF. Supplementary information includes the following information: (1) PRISMA checklist (Online Resource 1); (2) search terms for each database (Online Resource 2); (3) characteristics of included studies (Online Resource 3); (4) quality assessment for all include studies (Online Resource 4) and (5) PROGRESS-plus equality domain reporting across all included studies (Online Resource 5)

## References

[1] World Health Organization (WHO) (2022) Cancer. Available from: https://www.who.int/news-room/fact-sheets/detail/cancer. Accessed 17 Oct 2022

[2] Cancer Research UK (2014) Cancer survival statistics. Available from: https://www.cancerresearchuk.org/health-professional/cancer-statistics/survival/all-cancers-combined. Accessed 17 Oct 2022

[3] Schaapveld M (2008). Risk of new primary nonbreast cancers after breast cancer treatment: a Dutch population-based study. J Clin Oncol.

[4] Strongman H (2019). Medium and long-term risks of specific cardiovascular diseases in survivors of 20 adult cancers: a population-based cohort study using multiple linked UK electronic health records databases. Lancet.

[5] Gerritsen JK, Vincent AJ (2016). Exercise improves quality of life in patients with cancer: a systematic review and meta-analysis of randomised controlled trials. Br J Sports Med.

[6] McTiernan A (2019). Physical activity in cancer prevention and survival: a systematic review. Med Sci Sports Exerc.

[7] Cancer Research UK (2019) Exercise guidelines for cancer patients. Available from: https://www.cancerresearchuk.org/about-cancer/coping/physically/exercise-guidelines. Accessed 6 Oct 2022

[8] Chung JY (2013). Patterns of physical activity participation across the cancer trajectory in colorectal cancer survivors. Support Care Cancer.

[9] Irwin ML (2009). Physical activity interventions for cancer survivors. Br J Sports Med.

[10] Midgley AW (2018). Exercise program design considerations for head and neck cancer survivors. Eur Arch Otorhinolaryngol.

[11] Blanchard CM, Courneya KS, Stein K (2008). Cancer survivors' adherence to lifestyle behavior recommendations and associations with health-related quality of life: results from the American Cancer Society's SCS-II. J Clin Oncol.

[12] Rogers LQ (2006). Physical activity and quality of life in head and neck cancer survivors. Support Care Cancer.

[13] O'Donoghue G (2018). Socio-economic determinants of physical activity across the life course: a "DEterminants of DIet and Physical ACtivity" (DEDIPAC) umbrella literature review. PLoS ONE.

[14] Nguyen J-M (2021). Interaction between physical activity and socioeconomic determinants among cancer patients: a systematic mapping review. J Cancer Sci Clin Ther.

[15] Hanbury A, Wood H (2018). Using behavioural science to explore patient perceptions. Int J Pharm Healthcare Mark.

[16] Clifford BK (2018). Barriers and facilitators of exercise experienced by cancer survivors: a mixed methods systematic review. Support Care Cancer.

[17] Fox L (2019). Barriers and facilitators to physical activity in men with prostate cancer: a qualitative and quantitative systematic review. Psychooncology.

[18] Lavallée JF (2019). Barriers and facilitators to participating in physical activity for adults with breast cancer receiving adjuvant treatment: a qualitative metasynthesis. Psychooncology.

[19] Romero-Elías M (2017). Factors that promote or hinder physical activity participation in patients with colorectal cancer: a systematic review. Psychol Soc Educ.

[20] Michie S, van Stralen MM, West R (2011). The behaviour change wheel: a new method for characterising and designing behaviour change interventions. Implement Sci.

[21] Michie S, Atkins L, West R (2014) *The behaviour change wheel*: A guide to designing interventions. London: Silverback Publishing. http://www.behaviourchangewheel.com

[22] Cane J, O’Connor D, Michie S (2012). Validation of the theoretical domains framework for use in behaviour change and implementation research. Implement Sci.

[23] Michie S (2005). Making psychological theory useful for implementing evidence based practice: a consensus approach. Qual Saf Health Care.

[24] Moher D (2009). Preferred reporting items for systematic reviews and meta-analyses: the PRISMA statement. PLoS Med.

[25] Caspersen CJ, Powell KE, Christenson GM (1985). Physical activity, exercise, and physical fitness: definitions and distinctions for health-related research. Public Health Rep.

[26] Bronfenbrenner U (1977). Toward an experimental ecology of human development. Am Psychol.

[27] Hong QN (2018). The Mixed Methods Appraisal Tool (MMAT) version 2018 for information professionals and researchers. Educ Inf.

[28] Critical Appraisal Skills Programme (CASP) (2018) Critical Appraisal Skills Programme (CASP) Qualitative Studies Checklist. Available from: https://casp-uk.net/casp-tools-checklists/. Accessed 3 June 2022

[29] O'Neill J (2014). Applying an equity lens to interventions: using PROGRESS ensures consideration of socially stratifying factors to illuminate inequities in health. J Clin Epidemiol.

[30] Thomas J, Harden A (2008). Methods for the thematic synthesis of qualitative research in systematic reviews. BMC Med Res Methodol.

[31] Ning Y (2022). Barriers and facilitators to physical activity participation in patients with head and neck cancer: a scoping review. Support Care Cancer.

[32] Brunet J (2013). A qualitative exploration of barriers and motivators to physical activity participation in women treated for breast cancer. Disabil Rehabil.

[33] Cheung DST (2021). Exercise levels and preferences on exercise counselling and programming among older cancer survivors: a mixed-methods study. J Geriatr Oncol.

[34] Craike MJ (2011). An exploratory study of the factors that influence physical activity for prostate cancer survivors. Support Care Cancer.

[35] Cummins C (2017). Navigating physical activity engagement following a diagnosis of cancer: a qualitative exploration. Eur J Cancer Care (Engl).

[36] DeGuzman PB, Chu C, Keim-Malpass J (2019). Built and Natural environment barriers and facilitators to physical activity in rural, suburban, and small urban neighborhoods. Oncol Nurs Forum.

[37] Falzon C (2012). Beliefs about physical activity in sedentary cancer patients: an in-depth interview study in France. Asian Pac J Cancer Prev.

[38] Gentili C (2019). Body image issues and attitudes towards exercise amongst men undergoing androgen deprivation therapy (ADT) following diagnosis of prostate cancer. Psychooncology.

[39] Henriksson A (2016). Perceived barriers to and facilitators of being physically active during adjuvant cancer treatment. Patient Educ Couns.

[40] Keogh JW (2014). Perceived barriers and facilitators to physical activity in men with prostate cancer: possible influence of androgen deprivation therapy. Eur J Cancer Care (Engl).

[41] Larsson IL (2008). Women's experience of physical activity following breast cancer treatment. Scand J Caring Sci.

[42] Loh SY, Chew SL, Lee SY (2011). Physical activity and women with breast cancer: insights from expert patients. Asian Pac J Cancer Prev.

[43] Owusu C (2018). Perspective of older African-American and Non-Hispanic white breast cancer survivors from diverse socioeconomic backgrounds toward physical activity: a qualitative study. J Geriatr Oncol.

[44] Patel A, Schofield GM, Keogh JW (2021). Barriers to physical activity in prostate cancer survivors. N Z Med J.

[45] Renman D (2022). Attitudes to and experiences of physical activity after colon cancer diagnosis amongst physically active individuals - a qualitative study. Cancer Control.

[46] Rogers SN, Lowe D, Midgley AW (2022). Patients’ views of physical activity whilst living with and beyond head and neck cancer. Int J Oral Maxillofac Surg.

[47] Sander AP (2012). Factors that affect decisions about physical activity and exercise in survivors of breast cancer: a qualitative study. Phys Ther.

[48] Sealy MJ (2021). Perception and performance of physical activity behavior after head and neck cancer treatment: exploration and integration of qualitative and quantitative findings. Int J Environ Res Public Health.

[49] Smith L (2017). Cancer survivors' attitudes towards and knowledge of physical activity, sources of information, and barriers and facilitators of engagement: a qualitative study. Eur J Cancer Care (Engl).

[50] Smith SA (2017). Community engagement to address socio-ecological barriers to physical activity among African American breast cancer survivors. J Ga Public Health Assoc.

[51] Treviño RA (2012). Mexican-American and Puerto Rican breast cancer survivors' perspectives on exercise: similarities and differences. J Immigr Minor Health.

[52] Whitehead S, Lavelle K (2009). Older breast cancer survivors' views and preferences for physical activity. Qual Health Res.

[53] Williams F (2017). Physician role in physical activity for African-American males undergoing radical prostatectomy for prostate cancer. Support Care Cancer.

[54] Kim S (2020). The experience of cancer-related fatigue, exercise and exercise adherence among women breast cancer survivors: Insights from focus group interviews. J Clin Nurs.

[55] Avancini A (2021). Nurses' perspectives on physical activity promotion in cancer patients: A qualitative research. Eur J Oncol Nurs.

[56] Fong AJ (2018). A qualitative analysis of oncology clinicians’ perceptions and barriers for physical activity counseling in breast cancer survivors. Support Care Cancer.

[57] Granger CL (2016). Barriers to translation of physical activity into the lung cancer model of care. A qualitative study of clinicians' perspectives. Ann Am Thorac Soc.

[58] Haussmann A (2018). What hinders healthcare professionals in promoting physical activity towards cancer patients? The influencing role of healthcare professionals' concerns, perceived patient characteristics and perceived structural factors. Eur J Cancer Care (Engl).

[59] Patel A, Schofield G, Keogh J (2018). Influences on health-care practitioners' promotion of physical activity to their patients with prostate cancer: a qualitative study. J Prim Health Care.

[60] Robertson L (2013). Promotion and support of physical activity among cancer survivors: a service provider perspective. Psychooncology.

[61] Roscoe CMP (2022). The role of physical activity in cancer recovery: an exercise practitioner's perspective. Int J Environ Res Public Health.

[62] Santa Mina D (2015). Enablers and barriers in delivery of a cancer exercise program: the Canadian experience. Curr Oncol.

[63] Shea KM, Urquhart R, Keats MR (2020). Physical activity and cancer care in the atlantic canadian provinces: an examination of provider beliefs, practices, resources, barriers, and enablers. J Cancer Educ.

[64] Smith-Turchyn J (2016). Physical activity and breast cancer: a qualitative study on the barriers to and facilitators of exercise promotion from the perspective of health care professionals. Physiother Can.

[65] Agasi-Idenburg CS (2020). “I am busy surviving” - views about physical exercise in older adults scheduled for colorectal cancer surgery. J Geriatr Oncol.

[66] Romero-Elías M (2020). Barriers to physical activity participation in colorectal cancer patients during chemotherapy treatment: a qualitative study. Eur J Oncol Nurs.

[67] Bea JW (2018). Physical activity among navajo cancer survivors: a qualitative study. Am Indian Alsk Native Ment Health Res.

[68] Morrow GR (2002). Fatigue associated with cancer and its treatment. Support Care Cancer.

[69] Shi Q (2011). Symptom burden in cancer survivors 1 year after diagnosis: a report from the American Cancer Society's Studies of Cancer Survivors. Cancer.

[70] Van Den Beuken-Van MH (2016). Update on prevalence of pain in patients with cancer: systematic review and meta-analysis. J Pain Symptom Manag.

[71] Sagen Å, Kåresen R, Risberg MA (2009). Physical activity for the affected limb and arm lymphedema after breast cancer surgery. A prospective, randomized controlled trial with two years follow-up. Acta Oncol.

[72] Romero SAD (2018). The association between fatigue and pain symptoms and decreased physical activity after cancer. Support Care Cancer.

[73] Midgley AW (2020). Should survivors of head and neck cancer be considered a distinct special population within the context of exercise prescription?. Br J Oral Maxillofac Surg.

[74] Bluethmann SM (2019). Physical activity in older cancer survivors: what role do multimorbidity and perceived disability play?. J Aging Phys Act.

[75] Kraus WE (2019). Physical activity, all-cause and cardiovascular mortality, and cardiovascular disease. Med Sci Sports Exerc.

[76] Hall LH (2022). Delivering brief physical activity interventions in primary care: a systematic review. Br J Gen Pract.

[77] Ramsey I (2022). Exercise counselling and referral in cancer care: an international scoping survey of health care practitioners’ knowledge, practices, barriers, and facilitators. Support Care Cancer.

[78] Hamilton SN (2021). Patient-reported outcome measures in patients undergoing radiotherapy for head and neck cancer. Support Care Cancer.

[79] Nadler M (2017). Oncology care provider perspectives on exercise promotion in people with cancer: an examination of knowledge, practices, barriers, and facilitators. Support Care Cancer.

[80] Atkins L (2017). A guide to using the Theoretical Domains Framework of behaviour change to investigate implementation problems. Implement Sci.

