# Supplementary material for: Barriers to and facilitators of physical activity in adults living with and beyond cancer, with special emphasis on head and neck cancer: a systematic review of qualitative and mixed methods studies
Source: Support Care Cancer. 2023 Jul 17;31(8):471. doi: 10.1007/s00520-023-07925-x (PMC10352410; doi:10.1007/s00520-023-07925-x)
Supplement: Supplementary file 1 — Supplementary file1 (DOCX 102 KB) Supplementary information can be found in the attached PDF. Supplementary information includes the following information: (1) PRISMA checklist (Online Resource 1); (2) search terms for each database (Online Resource 2); (3) characteristics of included studies (Online Resource 3); (4) quality assessment for all include studies (Online Resource 4) and (5) PROGRESS-plus equality domain reporting across all included studies (Online Resource 5) [file 520_2023_7925_MOESM1_ESM.docx]

**Online Resource 1** PRISMA Checklist

| **Section and Topic** | **Item #** | **Checklist item** | **Location where item is reported** |
| --- | --- | --- | --- |
| **TITLE** | | |  |
| Title | 1 | Identify the report as a systematic review. | Page 1 |
| **ABSTRACT** | | |  |
| Abstract | 2 | See the PRISMA 2020 for Abstracts checklist. | Page 3 |
| **INTRODUCTION** | | |  |
| Rationale | 3 | Describe the rationale for the review in the context of existing knowledge. | Page 4 |
| Objectives | 4 | Provide an explicit statement of the objective(s) or question(s) the review addresses. | Page 4 and 5 |
| **METHODS** | | |  |
| Eligibility criteria | 5 | Specify the inclusion and exclusion criteria for the review and how studies were grouped for the syntheses. | Page 5 and 6 |
| Information sources | 6 | Specify all databases, registers, websites, organisations, reference lists and other sources searched or consulted to identify studies. Specify the date when each source was last searched or consulted. | Page 5 |
| Search strategy | 7 | Present the full search strategies for all databases, registers and websites, including any filters and limits used. | Online Resource 2 |
| Selection process | 8 | Specify the methods used to decide whether a study met the inclusion criteria of the review, including how many reviewers screened each record and each report retrieved, whether they worked independently, and if applicable, details of automation tools used in the process. | Page 5 |
| Data collection process | 9 | Specify the methods used to collect data from reports, including how many reviewers collected data from each report, whether they worked independently, any processes for obtaining or confirming data from study investigators, and if applicable, details of automation tools used in the process. | Page 5 |
| Data items | 10a | List and define all outcomes for which data were sought. Specify whether all results that were compatible with each outcome domain in each study were sought (e.g. for all measures, time points, analyses), and if not, the methods used to decide which results to collect. | Page 5 and 6 |
|  | 10b | List and define all other variables for which data were sought (e.g. participant and intervention characteristics, funding sources). Describe any assumptions made about any missing or unclear information. | Page 6 and 7 |
| Study risk of bias assessment | 11 | Specify the methods used to assess risk of bias in the included studies, including details of the tool(s) used, how many reviewers assessed each study and whether they worked independently, and if applicable, details of automation tools used in the process. | Page 6 |
| Effect measures | 12 | Specify for each outcome the effect measure(s) (e.g. risk ratio, mean difference) used in the synthesis or presentation of results. | N/A |
| Synthesis methods | 13a | Describe the processes used to decide which studies were eligible for each synthesis (e.g. tabulating the study intervention characteristics and comparing against the planned groups for each synthesis (item #5)). | Page 7 and 21 |
|  | 13b | Describe any methods required to prepare the data for presentation or synthesis, such as handling of missing summary statistics, or data conversions. | N/A |
|  | 13c | Describe any methods used to tabulate or visually display results of individual studies and syntheses. | Page 6 |
|  | 13d | Describe any methods used to synthesize results and provide a rationale for the choice(s). If meta-analysis was performed, describe the model(s), method(s) to identify the presence and extent of statistical heterogeneity, and software package(s) used. | N/A |
|  | 13e | Describe any methods used to explore possible causes of heterogeneity among study results (e.g. subgroup analysis, meta-regression). | N/A |
|  | 13f | Describe any sensitivity analyses conducted to assess robustness of the synthesized results. | N/A |
| Reporting bias assessment | 14 | Describe any methods used to assess risk of bias due to missing results in a synthesis (arising from reporting biases). | N/A |
| Certainty assessment | 15 | Describe any methods used to assess certainty (or confidence) in the body of evidence for an outcome. | N/A |
| **RESULTS** | | |  |
| Study selection | 16a | Describe the results of the search and selection process, from the number of records identified in the search to the number of studies included in the review, ideally using a flow diagram. | Page 22 |
|  | 16b | Cite studies that might appear to meet the inclusion criteria, but which were excluded, and explain why they were excluded. | Page 22 |
| Study characteristics | 17 | Cite each included study and present its characteristics. | Online Resource 3 |
| Risk of bias in studies | 18 | Present assessments of risk of bias for each included study. | Online Resource 4 |
| Results of individual studies | 19 | For all outcomes, present, for each study: (a) summary statistics for each group (where appropriate) and (b) an effect estimate and its precision (e.g. confidence/credible interval), ideally using structured tables or plots. | N/A |
| Results of syntheses | 20a | For each synthesis, briefly summarise the characteristics and risk of bias among contributing studies. | Page 7 |
|  | 20b | Present results of all statistical syntheses conducted. If meta-analysis was done, present for each the summary estimate and its precision (e.g. confidence/credible interval) and measures of statistical heterogeneity. If comparing groups, describe the direction of the effect. | Page |
|  | 20c | Present results of all investigations of possible causes of heterogeneity among study results. | N/A |
|  | 20d | Present results of all sensitivity analyses conducted to assess the robustness of the synthesized results. | N/A |
| Reporting biases | 21 | Present assessments of risk of bias due to missing results (arising from reporting biases) for each synthesis assessed. | N/A |
| Certainty of evidence | 22 | Present assessments of certainty (or confidence) in the body of evidence for each outcome assessed. | N/A |
| **DISCUSSION** | | |  |
| Discussion | 23a | Provide a general interpretation of the results in the context of other evidence. | Page 8-10 |
|  | 23b | Discuss any limitations of the evidence included in the review. | Page 10 |
|  | 23c | Discuss any limitations of the review processes used. | Page 10 |
|  | 23d | Discuss implications of the results for practice, policy, and future research. | Page 12 |
| **OTHER INFORMATION** | | |  |
| Registration and protocol | 24a | Provide registration information for the review, including register name and registration number, or state that the review was not registered. | Page 5 |
|  | 24b | Indicate where the review protocol can be accessed, or state that a protocol was not prepared. | Page 5 |
|  | 24c | Describe and explain any amendments to information provided at registration or in the protocol. | N/A |
| Support | 25 | Describe sources of financial or non-financial support for the review, and the role of the funders or sponsors in the review. | N/A |
| Competing interests | 26 | Declare any competing interests of review authors. | Page 13 |
| Availability of data, code and other materials | 27 | Report which of the following are publicly available and where they can be found: template data collection forms; data extracted from included studies; data used for all analyses; analytic code; any other materials used in the review. | N/A |

**Online Resource 2** Search terms for each database

**MEDLINE** Ovid MEDLINE(R)

1 exp Neoplasms/

2 cancer*.ti,ab,kw.

3 carcinoma*.ti,ab,kw.

4 neoplas*.ti,ab,kw.

5 tumor*.ti,ab,kw.

6 tumour*.ti,ab,kw.

7 malignan*.ti,ab,kw.

8 oncolog*.ti,ab,kw.

9 radiotherap*.ti,ab,kw.

10 chemotherap*.ti,ab,kw.

11 exp Radiotherapy/

12 1 or 2 or 3 or 4 or 5 or 6 or 7 or 8 or 9 or 10 or 11

13 exp Exercise/

14 exp Yoga/

15 (gym or yoga or circuit training).ti,ab,kw.

16 exercis*.ti,ab,kw.

17 physical activit*.ti,ab,kw.

18 physical fitness.ti,ab,kw.

19 physical exertion.ti,ab,kw.

20 resistance training.ti,ab,kw.

21 aerobic* training.ti,ab,kw.

22 13 or 14 or 15 or 16 or 17 or 18 or 19 or 20 or 21

23 12 and 22

24 barrier*.ti,ab,kw.

25 facilitator*.ti,ab,kw.

26 enabl*.ti,ab,kw.

27 hinder*.ti,ab,kw.

28 motivat*.ti,ab,kw.

29 exp Patient Compliance/

30 adhere.ti,ab,kw.

31 exp "Treatment Adherence and Compliance"/

32 (comply or compliance).ti,ab,kw.

33 engage*.ti,ab,kw.

34 obstacle*.ti,ab,kw.

35 restrain*.ti,ab,kw.

36 (inhibit or inhibits or inhibited).ti,ab,kw.

37 impede*.ti,ab,kw.

38 constrain*.ti,ab,kw.

39 hindrance*.ti,ab,kw.

40 exp Patient Acceptance of Health Care/

41 or/24-40

42 23 and 41

43 ((feasibil* or accept*) adj3 (exercis* or physical)).ti,ab,kw.

44 12 and 43

45 42 or 44

46 exp animals/

47 human/

48 46 not 47

49 45 not 48

50 limit 49 to english language

51 letter.pt.

52 editorial.pt.

53 comment.pt.

54 case reports.pt.

55 review.pt.

56 clinical conference.pt.

57 51 or 52 or 53 or 54 or 55 or 56

58 50 not 57 Limit to 2005-

**CINAHL Plus** EbscoHost

S1 (MH "Neoplasms+")

S2 TI cancer* OR AB cancer* OR SU cancer*

S3 TI carcinoma* OR AB carcinoma* OR SU carcinoma*

S4 TI neoplas* OR AB neoplas* OR SU neoplas*

S5 TI tumor* OR AB tumor* OR SU tumor*

S6 TI tumour* OR AB tumour* OR SU tumour*

S7 TI malignan* OR AB malignan* OR SU malignan*

S8 TI oncolog* OR AB oncolog* OR SU oncolog*

S9 TI radiotherap* OR AB radiotherap* OR SU radiotherap*

S10 TI chemotherap* OR AB chemotherap* OR SU chemotherap*

S11 (MH "Chemotherapy, Cancer+")

S12 (MH "Radiotherapy+")

S13 S1 OR S2 OR S3 OR S4 OR S5 OR S6 OR S7 OR S8 OR S9 OR S10 OR S11 OR S12

S14 (MH "Exercise+")

S15 (MH "Yoga+")

S16 TI exercis* OR AB exercis* OR SU exercis*

S17 TI (gym or yoga or circuit training) OR AB (gym or yoga or circuit training) OR SU (gym or yoga or circuit training)

S18 (MH "Physical Fitness+") OR (MH "Physical Activity")

S19 TI "physical activit*" OR AB "physical activit*" OR SU "physical activit*"

S20 TI "physical fitness" OR AB "physical fitness" OR SU "physical fitness"

S21 TI "physical exertion" OR AB "physical exertion" OR SU "physical exertion"

S22 TI "resistance training" OR AB "resistance training" OR SU "resistance training"

S23 TI "aerobic* training" OR AB "aerobic* training" OR SU "aerobic* training"

S24 S14 OR S15 OR S16 OR S17 OR S18 OR S19 OR S20 OR S21 OR S22 OR S23

S25 S13 AND S24

S26 TI barrier* OR AB barrier* OR SU barrier*

S27 TI facilitator* OR AB facilitator* OR SU facilitator*

S28 TI enabl* OR AB enabl* OR SU enabl*

S29 TI hinder* OR AB hinder* OR SU hinder*

S30 TI motivat* OR AB motivat* OR SU motivat*

S31 TI adhere OR AB adhere OR SU adhere

S32 TI ( (comply or compliance) ) OR AB ( (comply or compliance) ) OR SU ( (comply or compliance) ) OR (MH "Patient Compliance+")

S33 TI engage* OR AB engage* OR SU engage*

S34 TI obstacle* OR AB obstacle* OR SU obstacle*

S35 TI restrain* OR AB restrain* OR SU restrain*

S36 TI ( (inhibit or inhibits or inhibited) ) OR AB ( (inhibit or inhibits or inhibited) ) OR SU ( (inhibit or inhibits or inhibited) )

S37 TI impede* OR AB impede* OR SU impede*

S38 TI constrain* OR AB constrain* OR SU constrain*

S39 TI hindrance* OR AB hindrance* OR SU hindrance*

S40 (MH "Health Services Accessibility+")

S41 S26 OR S27 OR S28 OR S29 OR S30 OR S31 OR S32 OR S33 OR S34 OR S35 OR S36 OR S37 OR S38 OR S39 OR S40

S42 S25 AND S41

S43 TI ( ((feasibil* or accept*) N3 (exercis* or physical)) ) OR AB ( ((feasibil* or accept*) N3 (exercis* or physical)) ) OR SU ( ((feasibil* or accept*) N3 (exercis* or physical)) )

S44 S13 AND S43

S45 S42 OR S44

S46 S42 OR S44 Narrow by Language: - English

S47 PT book review OR PT commentary OR PT editorial OR PT letter

S48 S46 NOT S47 Limit to 2005-

**PsycINFO**

1 DE "Neoplasms" OR DE "Benign Neoplasms" OR DE "Breast Neoplasms" OR DE "Endocrine Neoplasms" OR DE "Leukemias" OR DE "Melanoma" OR DE "Metastasis" OR DE "Nervous System Neoplasms" OR DE "Terminal Cancer"

S2 TI cancer* OR AB cancer* OR KW cancer*

S3 TI carcinoma* OR AB carcinoma* OR KW carcinoma*

S4 TI neoplas* OR AB neoplas* OR KW neoplas*

S5 TI tumor* OR AB tumor* OR KW tumor*

S6 TI tumour* OR AB tumour* OR KW tumour*

S7 TI malignan* OR AB malignan* OR KW malignan*

S8 TI oncolog* OR AB oncolog* OR KW oncolog*

S9 TI radiotherap* OR AB radiotherap* OR KW radiotherap*

S10 TI chemotherap* OR AB chemotherap* OR KW chemotherap*

S11 DE "Chemotherapy"

S12 S1 OR S2 OR S3 OR S4 OR S5 OR S6 OR S7 OR S8 OR S9 OR S10 OR S11

S13 DE "Exercise" OR DE "Aerobic Exercise" OR DE "Weightlifting" OR DE "Yoga"

S14 TI exercis* OR AB exercis* OR KW exercis*

S15 TI (gym or yoga or circuit training) OR AB (gym or yoga or circuit training) OR KW (gym or yoga or circuit training)

S16 DE "Physical Fitness"

S17 DE "Physical Activity"

S18 TI "physical activit*" OR AB "physical activit*" OR KW "physical activit*"

S19 TI "physical fitness" OR AB "physical fitness" OR KW "physical fitness"

S20 TI "physical exertion" OR AB "physical exertion" OR KW "physical exertion"

S21 TI "resistance training" OR AB "resistance training" OR KW "resistance training"

S22 TI "aerobic* training" OR AB "aerobic* training" OR KW "aerobic* training"

S23 S13 OR S14 OR S15 OR S16 OR S17 OR S18 OR S19 OR S20 OR S21 OR S22

S24 S12 AND S23

S25 DE "Treatment Barriers"

S26 TI barrier* OR AB barrier* OR KW barrier*

S27 TI facilitator* OR AB facilitator* OR KW facilitator*

S28 TI enabl* OR AB enabl* OR KW enabl*

S29 TI hinder* OR AB hinder* OR KW hinder*

S30 TI motivat* OR AB motivat* OR KW motivat*

S31 DE "Motivation"

S32 TI adhere OR AB adhere OR KW adhere

S33 DE "Treatment Compliance"

S34 TI ( (comply or compliance) ) OR AB ( (comply or compliance) ) OR KW ( (comply or compliance) )

S35 TI engage* OR AB engage* OR KW engage*

S36 TI obstacle* OR AB obstacle* OR KW obstacle*

S37 TI restrain* OR AB restrain* OR KW restrain*

S38 TI ( (inhibit or inhibits or inhibited) ) OR AB ( (inhibit or inhibits or inhibited) ) OR KW ( (inhibit or inhibits or inhibited) )

S39 TI impede* OR AB impede* OR KW impede*

S40 TI constrain* OR AB constrain* OR KW constrain*

S41 TI hindrance* OR AB hindrance* OR KW hindrance*

S42 S25 OR S26 OR S27 OR S28 OR S29 OR S30 OR S31 OR S32 OR S33 OR S34 OR S35 OR S36 OR S37 OR S38 OR S39 OR S40 OR S41

S43 S24 AND S42

S44 TI ( ((feasibil* or accept*) N3 (exercis* or physical)) ) OR AB ( ((feasibil* or accept*) N3 (exercis* or physical)) ) OR KW ( ((feasibil* or accept*) N3 (exercis* or physical)) )

S45 S12 AND S44

S46 S43 OR S45

S47 PZ letter OR PZ editorial OR PZ comment/reply

S48 S46 NOT S47

S49 S46 NOT S47 Narrow by Language: - English Limit to 2005-

**Cochrane**

#1 MeSH descriptor: [Neoplasms] explode all trees

#2 (cancer*):ti,ab,kw

#3 (carcinoma*):ti,ab,kw

#4 (neoplas*):ti,ab,kw

#5 (tumor*):ti,ab,kw

#6 (tumour*):ti,ab,kw

#7 (malignan*):ti,ab,kw

#8 (oncolog*):ti,ab,kw

#9 (radiotherap*):ti,ab,kw

#10 (chemotherap*):ti,ab,kw

#11 MeSH descriptor: [Radiotherapy] explode all trees

#12 {OR #1-#11}{Amireault, 2015 #592}

#13 MeSH descriptor: [Exercise] explode all trees

#14 MeSH descriptor: [Yoga] explode all trees

#15 ((gym or yoga or circuit training)):ti,ab,kw

#16 (exercis*):ti,ab,kw

#17 (physical NEXT activit*):ti,ab,kw

#18 ("physical fitness"):ti,ab,kw

#19 ("physical exertion"):ti,ab,kw

#20 ("resistance training"):ti,ab,kw

#21 (aerobic* NEXT training):ti,ab,kw

#22 {OR #13-#21}

#23 #12 AND #22

#24 (barrier*):ti,ab,kw

#25 (facilitator*):ti,ab,kw

#26 (enabl*):ti,ab,kw

#27 (hinder*):ti,ab,kw

#28 (motivat*):ti,ab,kw

#29 MeSH descriptor: [Patient Compliance] explode all trees

#30 (adhere):ti,ab,kw

#31 MeSH descriptor: [Treatment Adherence and Compliance] explode all trees

#32 (comply or compliance):ti,ab,kw

#33 (engage*):ti,ab,kw

#34 (obstacle*):ti,ab,kw

#35 (restrain*):ti,ab,kw

#36 (inhibit or inhibits or inhibited):ti,ab,kw

#37 (impede*):ti,ab,kw

#38 (constrain*):ti,ab,kw

#39 (hindrance*):ti,ab,kw

#40 MeSH descriptor: [Patient Acceptance of Health Care] explode all trees

#41 {OR #24-#40}

#42 #23 AND #41

#43 ((feasibil* or accept*) NEAR/3 (exercis* or physical)):ti,ab,kw

#44 #12 AND #43

#45 #42 OR #44 Limit to 2005-

**Scopus**

[(TITLE-ABS-KEY ( neoplas* or cancer* or carcinoma* or tumor* or tumour* or malignan* or oncolog* or radiotherap* or chemotherap*)) AND (TITLE-ABS-KEY ( exercis* or gym or yoga or "circuit training" or "physical activit*" or "physical fitness" or "physical exertion" or "resistance training" or "aerobic* training" )) AND (TITLE-ABS- KEY ( barrier* or facilitator* or enabl* or hinder* or motivat* or adhere or comply or compliance or engage* or obstacle* or restrain* or inhibit or inhibits or inhibited or impede* or constrain* or hindrance*)) OR (TITLE-ABS-KEY ( neoplas* OR cancer* OR carcinoma* OR tumor* OR tumour* OR malignan* OR oncolog* OR radiotherap* OR chemotherap*)) AND (TITLE-ABS-KEY (( feasibil* OR accept* ) W/3 ( exercis* OR physical)))] AND

(LIMIT-TO (language, "english")) and Subject area (Medicine, Biochemistry, Genetics and Molecular Biology, Nursing, Psychology, Health Professions, Neuroscience, Social Sciences, Immunology and Microbiology)

Limit to 2005-

**Online Resource 3** Characteristics of included studies (n = 36)

| **Study** | **Aim** | **Sample size** | **Population** | **Outcome measure** | **Type of activity** | **Cancer Type** | **Length of time from diagnosis** | **Country** |
| --- | --- | --- | --- | --- | --- | --- | --- | --- |
| 65 | To investigate the barriers to, facilitators of, and preferences for exercise in the preoperative phase among older patients who are or were eligible for surgery for colorectal cancer, and their informal caregivers. To explore the views of health care professionals who offer preoperative training for cancer patients | 15 patients, 13 caregivers and nine healthcare professionals | Patients, caregivers, healthcare professionals | Interviews | Physical activity participation and promotion | Colorectal | Pre- and post- treatment | The Netherlands |
| 55 | This study aimed to qualitatively examine the perspectives of nurses about physical activity in cancer patients (according to the Theory of Planned Behaviour) | 14 (non-oncology) nurses | Healthcare professionals | Focus groups | Physical activity promotion | Healthcare professionals working with a variety of cancer types | N/A | Italy |
| 67 | This study aimed to assess Navajo cancer survivors physical activity habits and potential barriers to engaging in adequate physical activity. This study also evaluated physical activity preferences | 40 (32 cancer patients and 8 family members/friends) | Patients and family members/friends | Focus groups and interviews | Physical activity participation | Variety of cancer types (majority with colorectal or breast cancer) | Post-treatment | United States |
| 32 | This study aimed to explore breast cancer survivors’ perceptions of the factors influencing their ability to maintain self-directed physical activity, the factors breast cancer survivors identify as enabling their ability to maintain participation in physical activity and the factors they identify as hindering their ability to maintain participation in physical activity | 9 cancer patients | Patients | Interviews | Physical activity participation | Breast | Post-treatment | Canada |
| 33 | This study aimed to report the exercise levels of older cancer survivors, to assess and explore their exercise counselling and programming preferences, and to examine the correlates of exercise levels and interest in exercise counselling and programming | 290 patients (questionnaires); 12 patients (interviews) | Patients | Questionnaires and interviews | Physical activity participation | Variety of cancer types (most common cancers: colorectal, lung, breast, prostate) | Post-treatment | Hong Kong |
| 34 | To gain an understanding of the factors that influence participation in physical activity for survivors of prostate cancer and to examine changes in participation in physical activity pre- and post-diagnosis | 18 patients | Patients | Questionnaire and interviews | Physical activity | Prostate | Post- treatment | Australia |
| 35 | This study aimed to explore perspectives of cancer survivors regarding the barriers and facilitators they experience in undertaking physical activity following a diagnosis of cancer | 25 patients | Patients | Interviews | Physical activity participation | Variety of cancer types (most common cancer: breast) | Post-treatment | New Zealand |
| 36 | This study aimed to explore built and natural environment barriers and facilitators to walking for exercise in cancer survivors | 7 patients | Patients | Interviews (and photovoice) | Physical activity participation | Breast and kidney | During and post-treatment | United States |
| 37 | This study aimed to characterize the beliefs of cancer patients about physical activity, and identify the role of psychological barriers and stereotypes in these beliefs | 20 patients | Patients | Interviews | Physical activity participation | Variety of cancer types (most common cancers: breast, prostate, colorectal) | During-treatment | France |
| 56 | To examine factors affecting physical activity counselling in clinicians, and to understand potential clinician-based strategies that facilitate physical activity counselling | 27 healthcare professionals | Healthcare professionals | Focus groups | Physical activity promotion | Breast | N/A | Canada |
| 57 | This study aimed to identify factors (barriers and enablers) that influence clinicians’ translation of the physical activity guidelines into practice | 17 healthcare professionals | Healthcare professionals | Focus group and interviews | Physical activity promotion | Healthcare professionals working predominantly with lung cancer patients | N/A | Australia |
| 38 | This study aimed to explore the impact of Androgen Deprivation Therapy (ADT) side-effects on prostate cancer patients’ body image and sense of masculinity. This study also aimed to investigate patients’ attitudes towards exercise and potential exercise barriers in relation to ADT side-effects | 20 patients | Patients | Interviews | Physical activity participation | Prostate | During- and post-treatment | United Kingdom |
| 58 | To gain a comprehensive description of concerns, perceived patient characteristics and structural factors influencing healthcare professionals physical activity promotion and at identifying the reasons and mechanisms behind them | 30 healthcare professionals | Healthcare professionals | Interviews | Physical activity promotion | Breast, prostate and colorectal cancer | N/A | Germany |
| 39 | The aim was to describe cancer patients' perceived barriers and facilitators of physical activity during adjuvant cancer treatment | 23 patients | Patients | Individual interviews and focus groups | Physical activity participation | Breast, colorectal and prostate cancer | Pre- and during- treatment | Sweden |
| 40 | This study aimed to better understand the barriers and facilitators to physical activity in men with prostate cancer. This study also aimed to gaining preliminary insight into how ADT might influence these perceptions | 14 patients | Patients | Focus groups | Physical activity participation | Prostate | During- and post-treatment | Australia |
| 54 | This study explored cancer-related fatigue, barriers to exercise, perceived facilitative factors to overcome fatigue and implications for future exercise adherence programmes | 16 patients | Patients | Focus groups | Physical activity participation | Breast | Post-treatment | South Korea |
| 41 | This study aimed to describe how women treated for breast cancer experience physical activity after surgery | 12 patients | Patients | Interviews | Physical activity participation | Breast | Post-treatment | Sweden |
| 42 | To explore perception (myths, barriers and facilitators) of physical activity among women with breast cancer | Six patients completed treatment and eight patients undergoing treatment | Patients | Focus groups | Physical activity participation | Breast | During and post- treatment | Malaysia |
| 43 | This study aimed to explore the perspective of older breast cancer survivors from diverse racial and socioeconomic backgrounds toward physical activity, to inform the design of a physical activity program | 60 patients (60 patients took part in interview, 45 of these took part in follow-up focus groups) | Patients | Interviews and focus groups | Physical activity participation | Breast | Post-treatment | United States |
| 59 | This study aimed to identify factors that influenced healthcare practitioners to either promote or not promote physical activity to their prostate cancer patients | 16 healthcare professionals | Healthcare professionals | Interviews | Physical activity promotion | Healthcare professionals working with prostate cancer | N/A | New Zealand |
| 44 | To gain further insight regarding barriers to physical activity in older-aged prostate cancer survivors | 16 patients | Patients | Interviews | Physical activity participation | Prostate | Post- treatment | New Zealand |
| 45 | This study aimed to enlighten how the diagnosis of colorectal cancer might affect physically active individuals in their attitude and experiences towards physical activity | 20 patients | Patients | Interviews | Physical activity participation | Colorectal | Pre-treatment | Sweden |
| 60 | The aim of this study was to describe some of the current approaches used by the Cancer Society of New Zealand to support physical activity among survivors, barriers and facilitators associated with providing physical activity programmes and resources and barriers, facilitators and preferences regarding physical activity participation among cancer survivors | 17 healthcare professionals | Healthcare professionals | Interviews | Physical activity promotion | Healthcare professionals working with a variety of cancer types | N/A | New Zealand |
| 46 | The aim of this study was to give insight into head and neck cancer patients’ reflections on how and why they would be interested in participating in an exercise programme, including perceived ability to meet physical activity guidelines, perceived exercise benefits and barriers, and advice for others diagnosed with cancer | A stratified sample of 51 patients based on a previous survey, 25 took part in the interviews | Patients | Interviews to accompany survey data | Physical activity participation | Head and neck | Post- treatment | United Kingdom |
| 66 | This study aimed to explore the perceived disease-specific barriers to physical activity in colorectal cancer patients during adjuvant chemotherapy, including the perspectives of the patients, their relatives, and their health professionals | 30 participants (10 patients, 10 family members and 10 healthcare professionals) | Patients, family members and healthcare professionals | Interviews | Physical activity participation and promotion | Colorectal | During-treatment | Spain |
| 61 | This study aimed to explore the perceptions of exercise practitioners on the role of physical activity and the physiological and psychological benefits to recovering cancer patients. This study also aimed to understand the barriers and facilitators of promoting physical activity to cancer survivors | 5 healthcare professionals | Healthcare professionals | Interviews | Physical activity promotion | Healthcare professionals working with a variety of cancer types | N/A | Australia, United Kingdom, and Canada |
| 47 | This study aimed to investigate factors that affected decisions about physical activity and exercise in survivors of breast cancer and to determine whether fear was a factor | 34 patients | Patients | Focus groups and questionnaire data | Physical activity participation | Breast | During- and post-treatment | United States |
| 62 | This study aimed to examine existing cancer-exercise programs, experience with program development, and enablers and barriers to delivery | 14 healthcare professionals | Healthcare professionals | Interviews | Physical activity promotion | Healthcare professionals working with a variety of cancer types | N/A | Canada |
| 63 | This study aimed to describe available cancer-specific physical activity programs for cancer survivors in Atlantic Canada, to gain insight into the attitudes and practices if healthcare professionals and administrators with respect to physical activity and describe the barriers and enablers associated with implementing and maintaining these practices | 30 healthcare professionals | Healthcare professionals | Interviews | Physical activity promotion | Healthcare professionals working with a variety of cancer types | N/A | Canada |
| 48 | To explore head and neck cancer survivors’ views and experiences of physical activity, informed by the Attitude, Social Influence and Self-Effectiveness (ASE) model (including barriers and motivators to physical activity) and compare their self-reported physical activity levels to objectively measured levels of physical activity | 9 patients | Patients | Questionnaire, accelerometer data and interviews | Physical activity participation | Head and neck | Post- treatment | The Netherlands |
| 49 | The aim of the study was to assess attitudes towards and knowledge of physical activity, sources of information, and barriers and facilitators of engagement | 19 patients | Patients | Interviews | Physical activity participation | Variety of cancer types | Post- treatment | United Kingdom |
| 50 | This study aimed to explore barriers to physical activity, strategies for overcoming them, and intervention content and development | 60 patients | Patients | Focus groups | Physical activity participation | Breast | Post-treatment | United States |
| 64 | This study aimed to determine the barriers to and facilitators of exercise promotion by health care professionals for women with breast cancer | 24 healthcare professionals | Healthcare professionals | Interviews | Physical activity promotion | Healthcare professionals working with breast cancer patients | N/A | Canada |
| 51 | This study aimed to investigate the key variables that affect exercise adoption and maintenance, within specific cultural contexts of Hispanic cancer survivors, to gain their perspectives on the relevant issues surrounding breast cancer survivorship and exercise | 31 patients | Patients | Focus groups | Physical activity participation | Breast | Post-treatment | United States |
| 52 | This study aimed to explore the physical activity preferences of older female breast cancer survivors, to explore physical activity patterns before, during and post-illness. This study also examined physical activity motivators, facilitators, and barriers and views of and preferences for a tailored physical activity program | 29 patients | Patients | Interviews or focus group | Physical activity participation | Breast | Post-treatment | United Kingdom |
| 53 | To explore African American prostate cancer survivors’ experiences with physical activity prescription from their physicians, including views of barriers to physical activity | 12 patients | Patients | Focus groups | Physical activity participation and promotion | Prostate | During-treatment | United States |

**Online Resource 4** Quality assessment for all included studies (n = 36)

**Online Resource 4 (A)** Quality Assessment of Qualitative Studies (MMAT and CASP) (n = 30)

| **Study** | **SCREENING QUESTIONS** | | **MMAT QUALITATIVE STUDIES** | | | | | **CASP**  ***[Q4 Qualitative Studies Checklist]*** |
| --- | --- | --- | --- | --- | --- | --- | --- | --- |
|  | *Are there clear research questions?* | *Do the collected data allow to address the research questions?* | *Is the qualitative approach appropriate to answer the research question?* | *Are the qualitative data collection methods adequate to address the research question?* | *Are the findings adequately derived from the data?* | *Is the interpretation of results sufficiently substantiated by data?* | *Is there coherence between qualitative data sources, collection, analysis and interpretation?* | *Was the recruitment strategy appropriate to the aims of the research?* |
| 65 | Yes | Yes | Yes | Yes | Yes | Yes | Yes | Yes |
|  |  |  | In-depth interviews | The interview guide had open-questions and was developed iteratively with the theory of planned behaviour providing a framework to questioning | Combined content and thematic analysis. Open coding was completed, groups of codes were clustered to form themes using axial coding | The quotes used to justify the themes discussed are appropriate | There are clear links between data sources, collection, analysis, and interpretation. The authors clearly describe how the data has been collected, where it has been collected from and how it has been analysed and interpreted | Patients were recruited from the Netherlands Cancer Institute which is a recognized comprehensive cancer centre and a tertiary referral centre with a nationwide catchment area |
| 55 | Yes | Yes | Yes | Can’t tell | Yes | Yes | Yes | Yes |
|  |  |  | Focus groups | Topic guide was developed based on the literature and based on the theory of planned behaviour. However, the terms focus group and interviews are used interchangeably which makes it difficult to clearly determine which method was used. These  are separate qualitative methods and should be adequately differentiated | Interviews were analysed independently by two researchers using thematic analysis | The quotes used to justify the themes discussed are appropriate | There are clear links between data sources, collection, analysis, and interpretation. The authors clearly describe how the data has been collected, where it has been collected from and how it has been analysed and interpreted | Nurses were recruited by e-mail |
| 67 | Yes | Yes | Yes | Yes | Yes | Yes | Yes | Yes |
|  |  |  | Focus groups and interviews | The discussion guide incorporated theoretical frameworks to try and understand Navajo cancer survivor’s beliefs and barriers regarding cancer and physical activity | Audio recordings were used to document all focus groups and interview conversations, except for two participants where only field notes were taken based on participant preference. Field notes were integrated into the data when recordings were not available. A mixed deductive-inductive strategy was used to analyse transcripts | The quotes used to justify the themes discussed are appropriate | There are clear links between data sources, collection, analysis, and interpretation. The authors clearly describe how the data has been collected, where it has been collected from and how it has been analysed | Participants were recruited through flyers, direct contact by the Navajo oncology nurse on the research team, and by word of mouth |
| 32 | Yes | Yes | Yes | Yes | Yes | Yes | Yes | Yes |
|  |  |  | Interviews | An Associate Professor in physical activity psychology, developed a flexible semi-structured interview guide with the goal of exploring the factors that women felt affected their ability to maintain their physical activity participation | Data were analysed using thematic analysis, a semantic level analysis was conducted | The quotes used to justify the themes discussed are appropriate | There are clear links between data sources, collection, analysis, and interpretation. The authors clearly describe how the data has been collected, where it has been collected from and how it has been analysed | Eight of the participants were recruited through their health care providers who distributed information about the study during scheduled appointments. One woman was recruited by referral from another participant |
| 35 | Yes | Yes | Yes | Yes | Yes | Yes | Yes | Yes |
|  |  |  | Interviews | Data were collected using face to face, semi-structured interviews. Key areas of discussion included current levels of activity, how this compared to pre-diagnosis, experiences of physical activity engagement post-diagnosis and perspectives regarding what helped or hindered this process and makes it difficult to take part in physical activity | A thematic approach to data analysis was used | The quotes used to justify the themes discussed are appropriate | There are clear links between data sources, collection, analysis, and interpretation. The authors clearly describe how the data has been collected, where it has been collected from and how it has been analysed | The authors purposefully sought to include people who varied in terms of their current level of physical activity engagement including those who were not currently active in any way. Subsequently, theoretical sampling was used to further explore emerging findings. Recruitment strategies included fliers distributed via cancer support services, an editorial in a local newspaper, networking via personal and professional contacts, and snowballing |
| 36 | Yes | Yes | Yes | Yes | Yes | Yes | Yes | Yes |
|  |  |  | Interviews | Exploration of the built environment for walking may be viewed through a community lens. Therefore, the authors used a qualitative descriptive design with photovoice to explore cancer survivors’ experiences with walkability in their own neighbourhoods | Interview transcripts were analysed by the research team using a line-by-line inductive approach to analysis | The quotes used to justify the themes discussed are appropriate | There are clear links between data sources, collection, analysis, and interpretation. The authors clearly describe how the data has been collected, where it has been collected from and how it has been analysed | The authors recruited survivors from an outpatient cancer centre in Albemarle County and a community health fair in Charlottesville, both located in central Virginia. This area was chosen not only as a convenience sample but also because of the ability to recruit participants from small urban, suburban, and rural neighbourhoods |
| 37 | Yes | Yes | Yes | Yes | Yes | Yes | Yes | Yes |
|  |  |  | Interviews | The interview guide was developed based on previous research and beliefs about physical activity were integrated on the basis of previous work on the theory of planned behaviour | A content analysis was performed to analyse the transcripts of interviews. The two researchers discussed the categorization until a consensus was reached. A third researcher, also familiar with qualitative methods, then checked the consistency of the categories and subcategories | The quotes used to justify the themes discussed are appropriate | There are clear links between data sources, collection, analysis, and interpretation. The authors clearly describe how the data has been collected, where it has been collected from and how it has been analysed | Participants were recruited from a non-medical centre for cancer patients |
| 56 | Yes | Yes | Yes | Yes | Yes | Yes | Yes | Yes |
|  |  |  | Focus groups | A constructivist paradigm was used to understand experiences and views of clinicians who work directly with cancer patients. Understanding the interactions between clinicians and breast cancer survivors regarding physical activity was constructed socially between clinicians, and the interviewer through discussions during focus groups | Transcribed verbatim and inductive thematic analysis | The quotes used to justify the themes discussed are appropriate | There are clear links between data sources, collection, analysis, and interpretation. The authors clearly describe how the data has been collected, where it has been collected from and how it has been analysed and interpreted | To recruit participants, regional cancer centre vice presidents were contacted |
| 57 | Yes | Yes | Yes | Yes | Yes | Yes | Yes | Yes |
|  |  |  | Focus group and interviews | To address the research objectives, a qualitative study design employing focus group and one-on-one interviews were used. Two female PhD-qualified academic physical therapists conducted the focus group and interviews. One researcher had 14 years of experience in qualitative research methodology, including formal training, and provided training and mentorship to the second facilitator | All interviews were deidentified, transcribed verbatim, and independently cross-checked by a second researcher. Two researchers independently performed data coding and cross-checked thematic analyses | The quotes used to justify the themes discussed are appropriate | There are clear links between data sources, collection, analysis, and interpretation. The authors clearly describe how the data has been collected, where it has been collected from and how it has been analysed and interpreted | Healthcare professionals were recruited using purposive sampling from five hospitals in Melbourne, Victoria |
| 38 | Yes | Yes | Yes | Yes | Yes | Yes | Yes | Yes |
|  |  |  | Interviews | The interview topic guide was developed by the first author on the basis of previous literature and after discussing potential topics with patients and fellow researchers | The transcripts were analysed using NVivo, applying thematic analysis and constant comparative method. This approach is not guided by any pre‐existing theoretical framework and is therefore ideal for relatively unexplored fields | The quotes used to justify the themes discussed are appropriate | There are clear links between data sources, collection, analysis, and interpretation. The authors clearly describe how the data has been collected, where it has been collected from and how it has been analysed and interpreted | Participants were recruited through charities, social media, and press release |
| 58 | Yes | Yes | Yes | Yes | Yes | Yes | Yes | Yes |
|  |  |  | Interviews | Healthcare professionals were asked to counsel physical activity to a fictitious patient with breast or prostate cancer, mimicked by the interviewer, in the same way they would do it in their daily routine | Thematic analysis | The quotes used to justify the themes discussed are appropriate | There are clear links between data sources, collection, analysis, and interpretation. The authors clearly describe how the data has been collected, where it has been collected from and how it has been analysed and interpreted | Multiple recruitment strategies were pursued to take different working environments of healthcare professionals into account |
| 39 | Yes | Yes | Yes | Yes | Yes | Yes | Yes | Yes |
|  |  |  | Individual interviews and focus groups | Five women were interviewed individually as the authors outline it was not feasible to gather a focus group before the participants started their treatment because of the short interval between recruitment and commencing treatment | The interviews were transcribed verbatim and analysed according to Graneheim and Lundman’s guidelines for qualitative content analysis | The quotes used to justify the themes discussed are appropriate | There are clear links between data sources, collection, analysis, and interpretation. The authors clearly describe how the data has been collected, where it has been collected from and how it has been analysed and interpreted | Participants were recruited at a University Hospital in Sweden |
| 40 | Yes | Yes | Yes | Yes | No | Yes | yes | Yes |
|  |  |  | Focus groups | A semi-structured interview schedule involving seven probe questions was developed for the wider study based on the literature on the quality of life and determinants of physical activity in cancer survivors. Separate focus groups were arranged for the different groups of patients, each facilitated by the same research team members | The discussions held by each focus group were audio-taped and transcribed verbatim. The transcripts were analysed using an inductive thematic approach | The quotes used to justify the themes discussed are appropriate | There are clear links between data sources, collection, analysis, and interpretation. The authors clearly describe how the data has been collected, where it has been collected from and how it has been analysed and interpreted | Participants were recruited via urologists in private practice and a public hospital |
| 41 | Yes | Yes | Yes | Yes | Yes | Yes | Yes | Yes |
|  |  |  | Interviews | An interview guide was constructed consisting of semi-structured questions, starting with basic data, and then followed by questions within the area of physical activity, including received information | The transcribed material was analysed according to the procedure described by Dahlgren and Fahlsberg. The first four steps were analysed by the physiotherapists who had conducted the interviews, supervised by the first author with experience in phenomenological epistemology | The quotes used to justify the themes discussed are appropriate | There are clear links between data sources, collection, analysis, and interpretation. The authors clearly describe how the data has been collected, where it has been collected from and how it has been analysed and interpreted | Twelve women, treated for breast cancer, were identified through the Regional Tumour Registry in the South Sweden Region. They were strategically selected according to hospital, age and length of time since surgery. Three hospitals in the south of Sweden were chosen, one local, one regional and one University Hospital |
| 42 | Yes | Yes | Yes | Yes | No | Yes | No | Yes |
|  |  |  | Focus groups with women undergoing treatment, or who had completed treatment | The focus group topic guide consisted of a semi-structured question to cover their perceptions on physical activity and breast cancer, barriers, and facilitators to exercise. Three posters for promoting physical activity in relation to cancer risk prevention were used as visual props | Description of the analytical approach utilised was very brief, it is not clear how, for example, categories were established, or how conflicts were negotiated | The quotes used to justify the themes discussed are appropriate | The discussion section is not made clear. The results and discussion sections seem to be blended and the quotes are used in tandem with literature references | Participants randomly selected from the databases of new and old breast cancer cases |
| 43 | Yes | Yes | Yes | Yes | Yes | Can’t tell | Yes | Yes |
|  |  |  | Interviews and focus groups | Participants were interviewed one-on-one by one of two interviewers using open-ended and semi-structured questions. Approximately two months after interviews, preliminary analyses were completed after which participants were invited to participate in one of two follow-up focus groups led by a facilitator who had not participated in conducting the initial interviews. The goals were to; i) validate themes and conclusions derived from interviews and; ii) garner input from participants to design a physical activity program | Thematic analysis of the data was employed using a constant comparative analysis method | While the quotes used to substantiate the data are adequate, there are few quotes used for all of the themes, and the results do not seem very detailed | There are clear links between data sources, collection, analysis, and interpretation. The authors clearly describe how the data has been collected, where it has been collected from and how it has been analysed and interpreted | Participants were recruited from two local hospitals. Potentially eligible patients were identified from tumour registries, contacted by phone and informed about the study |
| 59 | Yes | Yes | Yes | Yes | Yes | Yes | Yes | Yes |
|  |  |  | Interviews | A structured interview schedule was developed from the literature. Questions were about the two main topic areas: (1) factors facilitating physical activity promotion by health-care practitioners to patients with prostate cancer; and (2) barriers to the promotion of physical activity | Data were analysed using an inductive thematic approach based on Auerbach and Silverstein’s theory. The first author transcribed all inter-views and identified initial themes | The quotes used to justify the themes discussed are appropriate | There are clear links between data sources, collection, analysis, and interpretation. The authors clearly describe how the data has been collected, where it has been collected from and how it has been analysed and interpreted | The authors purposively sampled oncologists, urologists, nurse specialists, general practitioners, physio-therapists and acupuncturists who provide treatment to patients with prostate cancer |
| 44 | Yes | Yes | Yes | Yes | Yes | Yes | Yes | Yes |
|  |  |  | Interviews | Questions were based on relevant literature relating to barriers to physical activity that prostate cancer patients can encounter | Interviews were transcribed verbatim and analysed using an inductive thematic approach | The quotes used to justify the themes discussed are appropriate | There are clear links between data sources, collection, analysis, and interpretation. The authors clearly describe how the data has been collected, where it has been collected from and how it has been analysed and interpreted | The majority of participants were recruited through the Prostate Cancer Foundation |
| 45 | Yes | Yes | Yes | Yes | Yes | Yes | Yes | Yes |
|  |  |  | Interviews | An interview guide was established after discussion between three of the authors. After the first three interviews, the authors went through the transcriptions to evaluate if the questions satisfied the aim of the study and whether the interviews were conducted satisfactorily. This resulted in the addition of an extra question regarding information of physical activity in the interview guide | Qualitative content analysis with an inductive approach according to Graneheim and Lundman was performed | The quotes used to justify the themes discussed are appropriate | There are clear links between data sources, collection, analysis, and interpretation. The authors clearly describe how the data has been collected, where it has been collected from and how it has been analysed and interpreted | Study participants were recruited from three healthcare regions in Northern Sweden |
| 60 | Yes | Yes | Yes | Yes | Yes | Yes | Yes | Yes |
|  |  |  | Semi-structured interviews | A pragmatic theoretical framework underpinned the work, allowing for the research to be led by the needs of the project | The analysis followed a pragmatic process of abduction, combining inductive and deductive approaches | The quotes used to justify the themes discussed are appropriate | There are clear links between data sources, collection, analysis, and interpretation. The authors clearly describe how the data has been collected, where it has been collected from and how it has been analysed and interpreted | Recruitment through the Cancer Society of New Zealand |
| 66 | Yes | Yes | Yes | Yes | Yes | Yes | Yes | Yes |
|  |  |  | Interviews | Semi-structured interviews were conducted with each participant and audio-recorded. Questions for patients, relatives, and health professionals were developed by members of the research team, who had expertise in qualitative research and physical activity promotion | The data were analysed with an inductive thematic analysis | The quotes used to justify the themes discussed are appropriate | There are clear links between data sources, collection, analysis, and interpretation. The authors clearly describe how the data has been collected, where it has been collected from and how it has been analysed and interpreted | Patients were recruited through the oncology unit of a Spanish hospital |
| 61 | Yes | Yes | Yes | Yes | Yes | Yes | Yes | Yes |
|  |  |  | Interviews | Data was collected via semi-structured, one-to-one interviews, which have been identified as effective in providing in-depth and insightful accounts from health professionals | Each interview, be that face-to-face or online, was digitally recorded and transcribed verbatim, reviewed for grammatical accuracy and re-read for familiarity by the lead researcher. Data was then analysed through a process of thematic analysis, guided by Braun and Clarke’s theory | The quotes used to justify the themes discussed are appropriate | There are clear links between data sources, collection, analysis, and interpretation. The authors clearly describe how the data has been collected, where it has been collected from and how it has been analysed and interpreted | A purposive sample of five cancer rehabilitation exercise practitioners. The participants were based in a variety of settings, for example, in local leisure centres and employed on a community basis. Participants’ country of work varied across different continents; the United Kingdom, Canada and Australia |
| 62 | Yes | Yes | Yes | Yes | Yes | Yes | Yes | Yes |
|  |  |  | Interviews | The study team developed an interview schedule comprised of structured and semi structured questions. The semi structured interview component used open-ended questions to investigate exercise program development, delivery, participation, strengths and weaknesses, and overall programmatic success. The interviews were pilot-tested twice with the study team to finalize question wording and sequencing | Inductive content analysis was used. Independent open coding of the initial 6 interview transcripts and then met to discuss and pursue agreement on codes and groupings. The same investigators then coded and grouped the remaining transcripts according to the initial transcript analyses, allowing for the addition of codes when appropriate. Upon completion of coding, the investigators met to identify categories and subcategories | The quotes used to justify the themes discussed are appropriate | There are clear links between data sources, collection, analysis, and interpretation. The authors clearly describe how the data has been collected, where it has been collected from and how it has been analysed and interpreted | An Internet search and review of Canadian publications in exercise intervention research for cancer patients was used to identify cancer exercise programmes. Snowball sampling of interview participants was used |
| 63 | Yes | Yes | Yes | Yes | Yes | Yes | Yes | Yes |
|  |  |  | Interviews | A semi-structured interview guide consisting of open and closed-ended questions was developed. Semi-structured questions were used to (1) identify the availability and describe the setting/ nature of cancer-specific physical activity programming available to survivors within the Atlantic provinces; (2) explore the attitudes of healthcare professionals and administrators with respect to physical activity for cancer survivors; (3) examine the practices of healthcare professionals with respect to physical activity discussions and counselling for cancer survivors; and (4) explore barriers and enablers of implementing existing cancer-specific physical activity programs | Interviews were analysed using an inductive constant comparative approach | The quotes used to justify the themes discussed are appropriate | There are clear links between data sources, collection, analysis, and interpretation. The authors clearly describe how the data has been collected, where it has been collected from and how it has been analysed and interpreted | Participants from cancer centres were interviewed. Snowball sampling was used by asking the initial group of informants to refer other individuals who may have knowledge of physical activity resources and programs for adult cancer survivors in their region or across the Atlantic provinces |
| 49 | Yes | Yes | Yes | Yes | Yes | Yes | Yes | Yes |
|  |  |  | Interviews | Interviews followed a topic guide developed by three researchers and informed by existing literature | Data were analysed using Thematic Analysis | The quotes used to justify the themes discussed are appropriate | There are clear links between data sources, collection, analysis, and interpretation. The authors clearly describe how the data has been collected, where it has been collected from and how it has been analysed and interpreted | This study was advertised on Cancer Research UK’s ‘Cancer Chat’ online forum and by posters and flyers displayed in the University College Hospital Macmillan Cancer Centre |
| 50 | Yes | Yes | Yes | Can’t tell | Can’t tell | Yes | No | Yes |
|  |  |  | Focus groups | Although focus groups were chosen for this study, there is a very short introduction and the aims/objectives are not clearly defined. As a result, it is unclear whether the qualitative methods adequately address the research question | There is little detail of the analytical process, including cross-checking/agreement on themes | Although presented in tables and not provided with much detail the quotes used to justify the themes discussed are appropriate | There is not enough detail in this paper to provide clear coherence between data sources, collection, analysis and interpretation | Participants were recruited from support groups |
| 64 | Yes | Yes | Yes | Yes | Yes | Yes | Yes | Yes |
|  |  |  | Interviews | One researcher conducted semi-structured interviews with participants. An interview guide was created on the basis of the study’s purpose and the results of a literature review. The interview guide was pilot tested on the first two participants, and minor revisions were made before implementing the final version | The analyses and reporting were performed according to conventional methods for descriptive qualitative studies. Discussions between the researchers occurred periodically during the transcription to consider how well the codes being developing addressed the research question | The quotes used to justify the themes discussed are appropriate | There are clear links between data sources, collection, analysis, and interpretation. The authors clearly describe how the data has been collected, where it has been collected from and how it has been analysed and interpreted | We recruited English-speaking HCPs who work with women with breast cancer who are treated as outpatients at cancer centres in southwestern Ontario. Participants were recruited through a combination of purposive and snowball sampling |
| 51 | Yes | Yes | Yes | Yes | Can’t tell | Yes | Yes | Yes |
|  |  |  | Focus groups | Participants were asked six questions: (1) ‘‘What comes to your mind when I say the word exercise?’’; (2) ‘‘What kind of exercise experiences have you had prior to cancer?” ; (3) ‘‘How do you think exercise might affect you in dealing with cancer?’’; (4) ‘‘Do you have any issues of safety or neighbourhood safety?’’ (5) ‘‘How do friends and family feel about exercise for themselves?’’ and ‘‘about you exercising?’’ (6) ‘‘Do you have any ideas about how to best deliver an exercise intervention within the Hispanic community?’’ Approximately four months after the initial session, the groups were reconvened. Participants were given a summary of the initial group session results and asked if anything was missed | The research team reviewed transcriptions from the group sessions and reached consensus for the emerging themes. However, there is no discussion of the analytical approach used | The quotes used to justify the themes discussed are appropriate | There are clear links between data sources, collection, analysis, and interpretation. The authors clearly describe how the data has been collected, where it has been collected from and how it has been analysed and interpreted | Focus group participants were recruited from an Oncology Hospital |
| 52 | Yes | Yes | Yes | Yes | Yes | Yes | Yes | Yes |
|  |  |  | Interviews or focus group | The interviews and focus groups took a semi structured format and examined current and previous physical activity patterns, physical activity motivators and facilitators, perceived and actual barriers, and preferences for a physical activity program targeted toward older breast cancer survivors. At the end of each session participants were asked if there was anything they would like to add | Thematic analysis was conducted | The quotes used to justify the themes discussed are appropriate | There are clear links between data sources, collection, analysis, and interpretation. The authors clearly describe how the data has been collected, where it has been collected from and how it has been analysed and interpreted | Participants were recruited from a number of breast cancer support groups in and around the cities of Manchester and Derby in the United Kingdom, and via a cancer charity based in Preston, also in the United Kingdom |
| 53 | Yes | Yes | Yes | Yes | Yes | Yes | Yes | Yes |
|  |  |  | Focus groups | A focus group was chosen as it was considered appropriate to elucidate discussion and interaction among this group of individuals to identify factors inductively | The questions were open ended, and comprised descriptive, probing, as well as opinion questions | The quotes used to justify the themes discussed are appropriate | There are clear links between data sources, collection, analysis, and interpretation. The authors clearly describe how the data has been collected, where it has been collected from and how it has been analysed and interpreted. | Participants were recruited from a larger observational cohort of prostate cancer patients |

**Online Resource 4 (B)** Quality Assessment of Mixed Methods Studies (MMAT and CASP) (n = 6)

| **Study** | **SCREENING QUESTIONS** | | **MMAT MIXED METHODS STUDIES** | | | | | **CASP**  ***[Q4 Qualitative Studies Checklist]*** |
| --- | --- | --- | --- | --- | --- | --- | --- | --- |
|  | *Are there clear research questions?* | *Do the collected data allow to address the research questions?* | *Is there an adequate rationale for using a mixed methods design to address the research question?* | *Are the different components of the study effectively integrated to answer the research question?* | *Are the outputs of the integration of qualitative and quantitative components adequately interpreted?* | *Are divergences and inconsistencies between quantitative and qualitative results adequately addressed?* | *Do the different components of the study adhere to the quality criteria of each tradition of the methods involved?* | *Was the recruitment strategy appropriate to the aims of the research?* |
| 33 | Yes | Yes | Yes | Yes | Yes | Yes | Yes | Yes |
|  |  |  | In the quantitative element, the exercise levels and preferences about exercise counselling and programming of older cancer survivors were examined. The qualitative element sought an in-depth understanding of how and why some participants viewed exercise positively while some had negative views | The qualitative and quantitative data are combined and synthesised effectively, tables and in-text results are summarised clearly | The authors have clearly outlined the reason for conducting in-depth interviews to accompany survey data and have displayed meta-inference by integrating both qualitative and quantitative data into their data analysis and discussion | There are no divergences or inconsistencies between qualitative and quantitative data discussed in this study | High criterion for both aspects | The recruitment was conducted in an oncology outpatient clinic of a public hospital in Hong Kong |
| 34 | Yes | Yes | Yes | Yes | Yes | Yes | Yes | Yes |
|  |  |  | The questionnaire gathered demographic and clinical information and physical activity levels, including current and pre-diagnosis participation | The majority of the data collected was in the form of qualitative data. However, the questionnaire data provided further information and context to the sample | The authors have described the importance of both qualitative and quantitative methodology and have displayed meta-inference by discussing them descriptively within the analysis and discussion | There are no divergences or inconsistencies between qualitative and quantitative data discussed in this study | High criterion for both aspects | Eligible participant details were obtained from two public and one private health service in Melbourne |
| 54 | Yes | Yes | Yes | Can’t tell | Yes | Yes | Yes | Yes |
|  |  |  | The questionnaire used gathered information about breast cancer patients’ fatigue levels | The majority of the data collected was in the form of qualitative data. However, the questionnaire quantitatively defined fatigue levels. The terms focus group and interviews are used interchangeably and although the method is described as *“focus group interviews”,* these methods are distinct and should be adequately differentiated | The authors have described the importance of both qualitative and quantitative methodology and have displayed meta-inference by discussing them descriptively within the analysis and discussion | There are no divergences or inconsistencies between qualitative and quantitative data discussed in this study | High criterion for both aspects | Convenience sampling was done on breast cancer survivors at Yonsei Medical Centre, Seoul, Korea. Flyers were posted in the hospital outpatient breast cancer clinic and radiotherapy centre |
| 46 | Yes | Yes | Yes | Yes | Yes | Yes | Yes | Yes |
|  |  |  | The rationale for the using this design was discussed as an aim: *“The aim of this study was to give more detail from the semi-structured telephone interviews that took place subsequent to the postal survey”* | For those selected for interviews, quantitative information from the survey accompanied the qualitative interview data | The authors have clearly outlined the reason for conducting semi-structured interviews to accompany survey data and have displayed meta-inference by integrating both qualitative and quantitative data into their data analysis and discussion | There are no divergences or inconsistencies between qualitative and quantitative data discussed in this study | High criterion for both aspects | A stratified sample of 51 patients based on age, gender and initial interest in an exercise programme were selected from 430 postal survey respondents |
| 47 | Yes | Yes | Yes | Can’t tell | Yes | Yes | Yes | Yes |
|  |  |  | This investigation was a grounded theory qualitative study in which focus groups and semi-structured interviews were used as the primary data collection methods. Qualitative data were triangulated in an analysis with data from two quantitative scales that measured participants’ beliefs about exercise and their activity levels. Triangulation provided a more comprehensive description of the participants in this study | The qualitative and quantitative results are clearly outlined in two separate headings in the results section, which provided a detailed description to participants. However, the terms focus group and interviews are used interchangeably and it is confusing as to which method was used. These methods are separate and should be adequately differentiated | The authors have clearly outlined the reason for collecting qualitative and quantitative data and have displayed meta-inference by integrating both qualitative and quantitative data into their data analysis and discussion | There are no divergences or inconsistencies between qualitative and quantitative data discussed in this study | High criterion for both aspects | Participants were recruited from a Midwestern medical centre through posted flyers, physicians, and other health care providers |
| 48 | Yes | Yes | Yes | Yes | Yes | Yes | Yes | Yes |
|  |  |  | The rationale for using a mixed methods design is clearly outlined. The aim was to compare patients qualitative data to their quantitative self-reported questionnaire data to objectively measured physical activity data | The qualitative and quantitative data are combined and synthesised effectively. These data are presented in tables and described in detail in-text | The authors have clearly outlined the reason for conducting a mixed methods study with self-reported questionnaires, objectively measured accelerometer data and semi-structured interviews. The authors have displayed meta-inference by integrating both qualitative and quantitative data into their data analysis and discussion | Any qualitative and quantitative data that was in contradiction to each other, is discussed by the authors | High criterion for both aspects | This study was a sub-study to the original research that compared daily step count to accelerometer data and collected self-reported questionnaire data. All participants invited to participate in this sub-study had to have participated in the prior study at time-point one (six to eight weeks post-treatment) |

**Online Resource 5** PROGRESS-Plus equality domain reporting across all included studies (n = 36)

| **Study** | **Place of residence**  ***(Environmental factors)*** | **Ethnicity** | **Occupation** | **Gender** | **Religion** | **Education *(Knowledge)*** | **Social capital** | **Socioeconomic Status *(Finance)*** | **+ Disability*** | **+ Age** | **+ Marital Status**  ***(Features of relationships)*** | **+ Time-dependent relationships**** |
| --- | --- | --- | --- | --- | --- | --- | --- | --- | --- | --- | --- | --- |
| 65 | C |  |  | C, A |  | C, A |  | A | C, A, D | C, A |  | C, A, D |
| 55 | C |  | C, A, D | C, A |  | A, D |  |  |  | A, D |  |  |
| 67 | C | C, A, D | D | C, A | C, A, D | A, D |  | A |  | C, A | A, D | C, A, D |
| 32 | C |  | A | C, A, D |  | A, D |  |  | A | A, D | A, D | C, A, D |
| 33 | C |  | A | C, A, D |  | C, A |  | C, A | C, A | C, A, D | C, A | C, A, D |
| 34 | C |  | C, A | C, A, D |  | C, A, D |  |  | C, A, D | C, A, D | C, A, D | C, A, D |
| 35 | C, A | C, A |  |  |  | A, D |  | A |  | C, A | A, D | C, A, D |
| 36 | C, A, D | D | A | C, A, D |  |  |  |  |  | C, A, D | D | C, A, D |
| 37 | C |  |  | C, A |  |  |  |  |  | C, A | A | C, A, D |
| 56 | C, A |  | C, A, D | C, A, D |  | C, A, D |  | A |  | A |  |  |
| 57 | C, A |  | C, A, D | C, A |  | C, A, D |  |  | A | C, A | A |  |
| 38 | C |  | A, D | C, A, D |  | C, A |  |  |  | A | C, A, D | C, A, D |
| 58 | C |  | C, A, D | C, A |  | C, A, D |  | A |  | A |  |  |
| 39 | C, A, D |  |  | C |  | C, A |  |  | C, A, D | C, A | A, D | C, A, D |
| 40 | C |  | A, D | C, A, D |  | A, D |  | D | A, D | C, A, D | A, D | C, A, D |
| 54 | C |  |  | C, A, D |  | A, D |  |  |  | C, A, D | A, D | C, A, D |
| 41 | C |  | A | C, A, D |  | D |  |  |  | C, A | A, D | C, A, D |
| 42 | C, A, D | C, A, D | C, A | C, A, D |  | C, A, D |  | C, A |  | C, A, D | C, A, D | C, A, D |
| 43 | C, A, D | C, A, D | A | C, A, D | A, D | C, A |  | C, A, D | A, D | C, A, D | A, D | C, A, D |
| 59 | C |  | C, A, D | C, A, D |  | A, D |  |  | D | C |  |  |
| 44 | C, A |  | A, D | C, A, D |  |  |  |  | A, D | C, A, D |  | C, A, D |
| 45 | C, A, D |  |  | C, A |  | A |  |  |  | C, A |  | C, A, D |
| 60 | C |  | C, A, D | A, D |  | A |  | A, D |  |  | A, D |  |
| 46 | C |  | A | C, A |  | D |  |  | C, A, D | C, A | A, D | C, A, D |
| 66 | C |  | C | C, A |  | D |  | D |  | C | A, D | C, A, D |
| 61 | C, A |  | C, A, D | C |  | A, D |  | A |  |  | A, D |  |
| 47 | C |  |  | C, A, D | C | A, D |  |  |  | C | A, D | C, A, D |
| 62 | C, A, D |  | C, A, D | C, A |  | C, A, D |  | A, D |  | C, A | A, D |  |
| 63 | C, A |  | C, A, D |  |  | A, D |  | A, D |  | C, A | A |  |
| 48 | C |  |  | C, A |  | C, D |  | A | A | C, A | A | C, A, D |
| 49 | C | C, A | C, A | C, A |  | C, A, D |  |  |  | C, A | C, A, D | C, A, D |
| 50 | C, A | C, A, D |  | C, A, D |  | A, D |  | A | A | C, A, D | A, D | C, A, D |
| 64 | C |  | C, A, D | C, A |  | A, D |  | A |  | A |  |  |
| 51 | C, A | C, A, D | C, A | C, A, D |  | A, D |  |  |  | C, A, D | C, A | C, A, D |
| 52 | C, A, D | C, D | A, D | C, A, D |  | A, D |  |  | A, D | C, A, D | A, D | C, A, D |
| 53 | C | C, A, D | C, A | C, A, D |  | C, A, D |  | C, A | A | C, A | C, A, D | C, A, D |

**For the purpose of this review, the PROGRESS-plus ‘disability’ characteristic related to factors that could impact on a patient’s physical function (for example, comorbidities).*

***The PROGRESS-plus characteristic ‘time-dependent relationships’ related to the length of time since diagnosis.*
